# γδ T cells shape memory-phenotype αβ T cell populations in non-immunized mice

**DOI:** 10.1371/journal.pone.0218827

**Published:** 2019-06-25

**Authors:** Swati Popat Phalke, Yafei Huang, Kira Rubtsova, Andrew Getahun, Deming Sun, Richard L. Reinhardt, Rebecca L. O’Brien, Willi K. Born

**Affiliations:** 1 Department of Biomedical Research, National Jewish Health, Denver, CO, United States of America; 2 Joint Laboratory for Stem Cell Engineering and Technology Transfer, School of Basic Medicine, Tongji Medical College, Huazhong University of Science and Technology, Wuhan, PR China; 3 Department of Immunology and Microbiology, University of Colorado Health Sciences Center, Aurora, CO, United States of America; 4 Doheny Eye Institute and Department of Ophthalmology, David Geffen School of Medicine at UCLA, Los Angeles, CA, United States of America; Rockefeller University, UNITED STATES

## Abstract

Size and composition of γδ T cell populations change dramatically with tissue location, during development, and in disease. Given the functional differentiation of γδ T cell subsets, such shifts might alter the impact of γδ T cells on the immune system. To test this concept, and to determine if γδ T cells can affect other immune cells prior to an immune response, we examined non-immunized mice derived from strains with different genetically induced deficiencies in γδ T cells, for secondary changes in their immune system. We previously saw extensive changes in pre-immune antibodies and B cell populations. Here, we report effects on αβ T cells. Similarly to the B cells, αβ T cells evidently experience the influence of γδ T cells at late stages of their pre-immune differentiation, as single-positive heat stable antigen-low thymocytes. Changes in these and in mature αβ T cells were most prominent with memory-phenotype cells, including both CD8+ and CD4+ populations. As previously observed with B cells, most of the effects on αβ T cells were dependent on IL-4. Unexpectedly, IL-4 seemed to be produced mainly by αβ T cells in the non-immunized mice, albeit strongly regulated by γδ T cells. Similarly to our findings with B cells, changes of αβ T cells were less pronounced in mice lacking all γδ T cells than in mice lacking only some, suggesting that the composition of the γδ T cell population determines the nature of the γδ-influence on the other pre-immune lymphocytes.

## Introduction

γδ T cell populations change in numbers and in cellular composition with human age [1], but also with infectious and autoimmune diseases [2]. In primary immune-deficiencies, the genetic background of an individual can be associated with such variations as well [2], and hematopoietic transplantation induces changes because γδ T cells are only slowly and incompletely restored [3, 4]. Likewise, mouse ontogeny is associated with an ordered appearance of γδ T cells expressing different TCR-Vγ genes (subsets), causing compositional shifts in the population over time. Furthermore, throughout life, different mouse tissues contain γδ T cell populations with distinct subset compositions [5, 6]. It was found that the TCR-Vγ-defined cell subsets have different functional potentials [7–12], suggesting a connection between innate TCR-ligand-specificities and functional differentiation [13–15], and overall changes of γδ T cell functions during ontogeny and between the tissues.

Albeit in small numbers, γδ T cells can exert a strong regulatory influence on inflammatory and immune responses [16, 17]. They also affect the immune system in the steady state [18]. Because of their functional differences, compositional shifts in the γδ T cell populations ought to alter the net-effect γδ T cells might have on other immune cells, but this influence has been difficult to demonstrate because external conditions that lead to shifts in γδ T cell populations often also directly affect other cell-types. Therefore, to specifically investigate the effect of compositional changes in γδ T cell populations on other cells, we assembled a collection of background-matched mice with genetic mutations in TCR genes causing the loss of some or all γδ T cells [19–21], as a model for the natural shifts in γδ T cell populations introduced above. These mice were not immunized, challenged, or treated in other ways. Nevertheless, their comparison revealed numerous secondary changes of the immune system, including differences in levels of immunoglobulin (Ig) subclasses [18, 22], in the composition of antibody specificities (repertoire) [18], in serum levels of cytokines and the capability of T cells to produce them [18], as well as shifts in granulocytes and B cells [18, 23].

We initially focused our investigation on the secondary changes in B cells, and confirmed that they were the result of interactions with the altered γδ T cell populations [18, 23]. The current study extends the investigation to secondary changes in αβ T cells. In the course of exploring underlying mechanisms, we compared effects on αβ T cells and B cells. As with B cells [18, 23], we found that changes in specific αβ T cell populations depend on the particular deficiency in γδ T cells. With either lymphocyte-type, the γδ-influence begins at late immature developmental stages, and most of the effects apparently are mediated by γδ T cell-regulated and αβ T cell-sourced interleukin 4 (IL-4).

## Materials and methods

### Mice

C57BL6J mice and γδ T cell-deficient mice of the same genetic background (B6.TCRδKO mice) were originally obtained from the Jackson Laboratory (Bar Harbor, ME) and bred at National Jewish Health (Denver, CO). TCR-Vγ4-/-/Vγ6-/- mice [20], which were a gift from Dr. Koichi Ikuta (Kyoto University, Kyoto, Japan) were then backcrossed onto the C57BL6J genetic background and re-established as homozygous line after 12 backcross generations (B6.TCR-Vγ4/6KO mice). B6.TCR-Vγ1-/- mice [24] were a gift from Dr. Simon Carding (Norwich Medical School, Norwich, U.K.), and provided by Dr. C. Wayne Smith (Baylor College of Medicine, Houston, TX). These mice were still further backcrossed onto the C57BL/6J genetic background and re-established as homozygous line after >10 backcross generations (B6.TCR-Vγ1KO mice). IL-4-/- mice (C57BL/6-^*Il4tm1Nnt*^/J) were obtained from the Jackson Laboratory and were a gift from Dr. P. Marrack at National Jewish Health. IL-4-/- mice were crossed with B6.TCR-Vγ4/6KO mice, double KO mice selected from the F2 generation and bred as a new homozygous line (B6.TCR-Vγ4/6KO/IL-4KO mice). All mice were cared for at National Jewish Health (NJH) following guidelines for normal and immune-deficient animals, and euthanized by CO2 inhalation following current guidelines of the American Veterinary Medical Association (AVMA). All efforts were made to minimize suffering. The experiments were conducted under a protocol approved by the National Jewish Health Institutional Animal Care and Use Committee (Protocol number AS2484).

Although the KO mice were backcrossed >10x, this approach cannot completely rule out an influence of genes closely linked to the locus of interest. Therefore, we sought to complement the genetic evidence with alternative ways of changing the same γδ T cell populations, using antibody treatment for γδ T cell-inactivation and adoptive cell-transfer for γδ T cell-reconstitution. Such experiments in our preceding studies confirmed that the altered γδ T cells mediate the changes in antibodies and B cells seen in the γδ-gene mutated mice [18, 22, 23, 25]. The similarity of γδ-effects on the αβ T-cells in this study, including changes seen with specific γδ-deficiencies, the critical role of IL-4, and the developmental timing of the γδ-effect on αβ T cells, suggest that similar mechanisms are at work, and that here as well interactions with γδ T cells are responsible for the effects.

### Flow cytometric analysis

Cells obtained from single cell suspensions (5 x 10^5^/well) were stained in 96-well plates (Falcon, BD Biosciences) for the cell surface markers shown in the figures, using the specific mAbs listed below. Lymphocytes were gated based on their forward (FSC) and side scatter (SC), and aggregates (doublets and other conjugates) were excluded by using forward scatter area (FSC-A) and forward scatter height (FSC-H). Samples were analyzed using LSR II and LSR Fortessa flow cytometers (Becton Dickinson). At least 1x10^5^ events were acquired per gated region, and the data were analyzed using FlowJo 10.5.2 software (TreeStar).

### Antibody reagents

Antibodies for flow cytometry included the following clones and conjugates: anti B220 (clone RA3-6B2, BV421, Biolegend, and APCCy7, eBiosciences); anti CD93 (clone AA4.1, APC, Invitrogen); anti CD23 (clone B3B4, PECy7, eBiosciences); anti IgM (clone B76, AF488, homemade); anti Fas (clone Jo2, PE, BD Pharmingen); anti IL-4Rα (clone mIL4R-M1, PE, BD Biosciences); anti CD3 (clone 17A2, PB, Biolegend); anti CD4 (clone RM4.5, APC, BD Biosciences and BV510, Biolegend); anti CD8β.2 (clone 53–5.8, FITC, BD Biosciences); anti Fas-L (clone mFL3, PE, eBiosciences); anti TCR-β (clone H57-597, PB, Biolegend and PercpCy5.5, eBiosciences); anti CD24 (clone M1/69, BV711, BD Biosciences); anti CD8α (clone 53–6.7, APCeF780, eBiosciences); anti CD122 (clone 5H4, PE, eBiosciences); anti CD44 (clone IM7, BV605, BD Biosciences); anti CD49d (clone R1-2, APC, Biolegend); anti CXCR3 (clone CXCR3-173, PECy7, Biolegend); anti T-bet (clone 4B10, BV421, Biolegend); anti Eomes (clone 1219A, AF488, R&D Systems).

### In vivo transient inactivation of T cells

In order to selectively inactivate T cells and subsets of T cells *in vivo*, we i.v.-injected affinity-purified mAbs specific for TCR-β (clone H57.597.2), TCR-δ (clone GL3), TCR-Vγ1 (clone 2.11) and TCR-Vγ4 (clone UC3) at 200 μg/mouse as previously described in detail [26]. This treatment does not deplete [27] but efficiently and specifically inactivates the targeted T cells (S1–S3 Figs) for up to two weeks [26]. The antibody-treated mice were used 7 days post injection for *in vivo* capture of IL-4.

### In vivo production of IL-4

*In vivo* production of IL-4 was measured using the Mouse IL-4 *In Vivo* Capture Assay Set (BD Pharmingen), following the method of Finkelman and Morris [28]. Unlike our earlier study [18], where we used an *in vivo* capture period of 8.5 hrs to demonstrate the difference in IL-4 production between C57BL/6 (wt) and B6.TCR-Vγ4/6KO mice, we extended the *in vivo* capture period here to 24 hrs. This modification increased the IL-4 signal roughly 2x to ~ 400 pg IL-4/ml serum but abolished the difference between the mouse strains, presumably because the injected capture antibody (10 μg/mouse) becomes limiting.

### Nomenclature

We used the nomenclature for murine TCR-Vγ genes introduced by Heilig and Tonegawa [29].

### Statistical analysis

Each experiment was carried out at least 3 times independently. Data are presented as means +/- SD. The One Way Nonparametric ANOVA (Kruskal Wallis) test was used for group comparisons. All data generated with γδ T cell-deficient mice were compared to the data generated with wt mice. A p value <0.05 was considered statistically significant.

## Results

### Splenic lymphopenia and loss of αβ T cells in mice lacking certain γδ T cells

γδ T cells encompass several subpopulations with different functional capabilities [8]. For example, the Vγ1+ γδ T cell population includes most of the γδ T cells capable of producing IL-4 [30], whereas the Vγ4+ and Vγ6+ populations harbor the vast majority of IL-17-producing γδ T cells [31, 32]. Because of these functional differences, shifts in the composition of total γδ T cells might be expected to change their overall functional “output” and consequentially, other cells and tissues.

**Fig 1:**
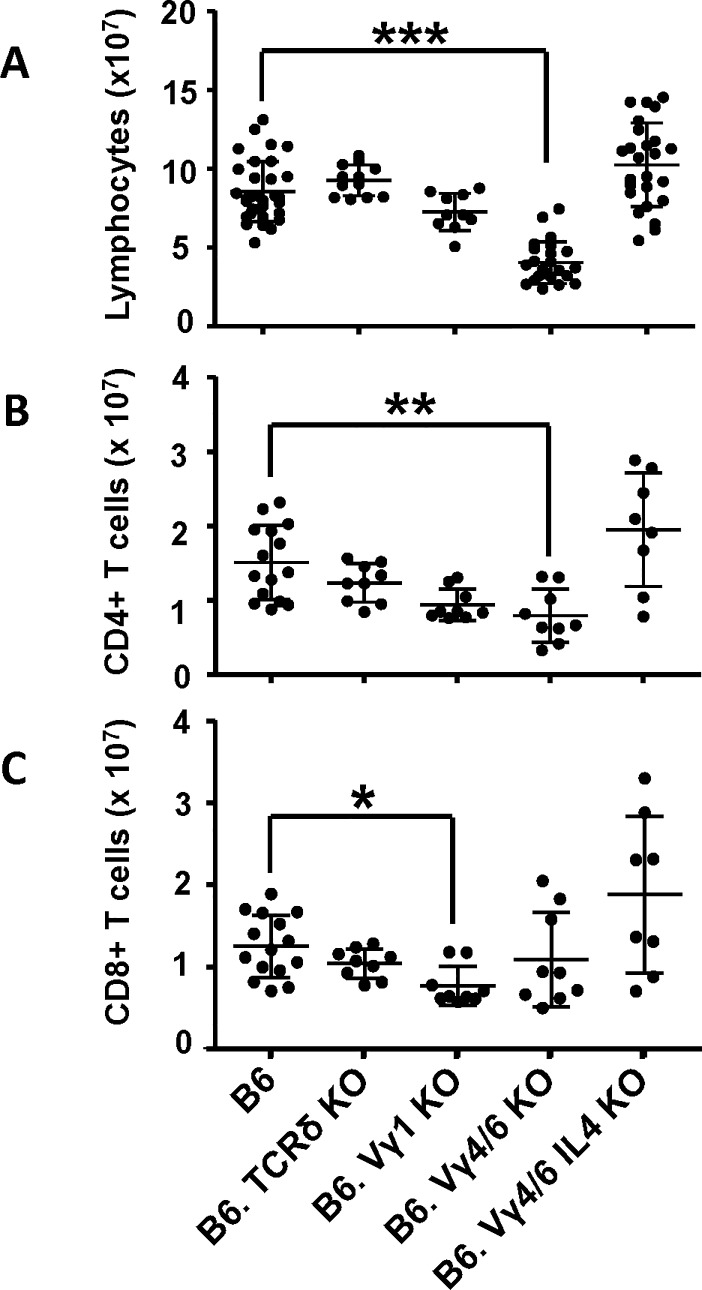
Deficiency in γδ T cells can affect splenic lymphocyte numbers and the size of splenic αβ T cell populations. A) Comparison of total numbers of lymphocytes/spleen in C57BL/6 (B6), B6.TCRδKO, B6.TCR-Vγ1KO, B6.TCR-Vγ4/6KO and B6.TCR-Vγ4/6KO/IL-4KO mice. n equal or greater than 10 mice/group. B) Numbers of CD4+ αβ T cells (TCR-β+CD4+ lymphocytes) in the spleen, in the same mouse strains as in A. n equal or greater than 8 mice/group. C) Numbers of CD8+ αβ T cells (TCR-β+CD8α+ lymphocytes) in the spleen, in the same mouse strains as in A. n equal or greater than 8 mice/group. Female and male mice ages 8–12 wks were included in the comparisons shown in Fig 1. *p<0.05, **p<0.01, ***p<0.001.

We compared C57BL/6 mice (wt) with background-matched mice deficient in all γδ T cells (B6.TCRδKO) or in only some γδ T cells, lacking Vγ1+ cells (B6.TCR-Vγ1KO) or lacking Vγ4+ and Vγ6+ cells (B6.TCR-Vγ4/6KO). In young adult mice (8–12 weeks old, male and female), the absence of all γδ T cells had little if any effect on splenic lymphocyte numbers (Fig 1A), or splenic αβ T cells (Fig 1B and 1C). However, the absence of Vγ1+ γδ T cells only was associated with significantly diminished numbers of CD8+ αβ T cells (Fig 1C). In contrast, the absence of Vγ4+ and Vγ6+ γδ T cells was associated with splenic lymphopenia (Fig 1A), and a pronounced loss of CD4+ but not of CD8+ αβ T cells (Fig 1B and 1C). These changes in splenic αβ T cell populations also affected their relative frequencies (S4 Fig). Of note, in terms of absolute numbers, the loss of αβ T cells in the spleen was much smaller than the previously reported loss of splenic B cells [23].

**Fig 2:**
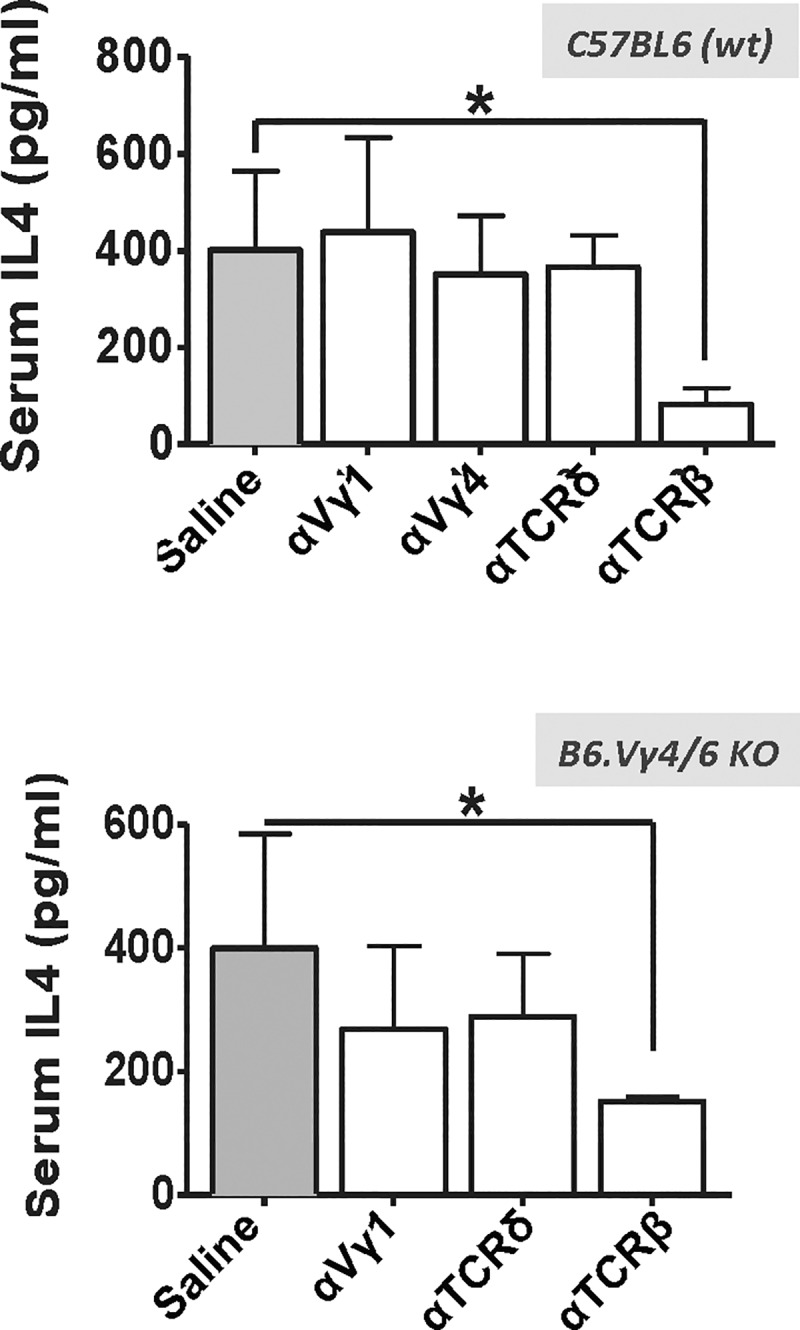
Contribution of T cells to serum IL-4 levels in non-immunized mice. Effect of *in vivo* treatment of C57BL/6 (wt) and B6.TCR-Vγ4/6KO mice with anti TCR mAbs on serum IL-4 levels: Mice were pre-treated with i.v. injections of saline or of the different TCR-specific mAbs specified in the figure, and serum IL-4 was later captured in vivo during a 24 hr period using an i.v. injected anti IL-4 mAb and subsequently detected by ELISA (see Methods). Top: C57BL/6 mice, n = 5; bottom: B6.TCR-Vγ4/6KO mice, n = 3. *p<0.05.

We previously investigated IL-4-production in non-immunized B6.TCR-Vγ4/6KO mice [18]. An *in vivo* cytokine capture assay [28], employed for a period of 8.5 hrs, indicated that these mice have higher IL-4 serum levels when compared to wt mice [18]. Their splenic T cells exhibited elevated IL-4 production following *in vitro* activation, and on a per cell basis, γδ T cells produced more IL-4 than αβ T cells. But γδ T cells in blood and spleen are less frequent than αβ T cells, and we did not resolve the relative contributions of the two T cell-types to overall IL-4 serum levels. To address this question, we again used the *in vivo* cytokine capture assay, but this time in combination with *in vivo* T cell inactivation by antibody treatment [26]. Firstly, we pre-treated the mice with i.v. injected TCR-V-specific mAbs, at doses previously shown to inactivate the targeted T cells for approximately two weeks [26]. All of the antibodies effectively targeted T cells (assessed on day 8 post injection), with no discernible effects on B cells (S1–S3 Figs). At one week into the window of T-inactivation, we secondly employed the *in vivo* cytokine capture assay. To achieve maximum sensitivity, we used a longer *in vivo* capture period (24 hrs), achieving roughly 2x greater accumulation of captured IL-4 by comparison with our earlier study [18]. However, we lost the ability to differentiate between wt control and B6.TCR-Vγ4/6KO mice, presumably because the injected capture antibody becomes limiting over the extended capture period. Indeed, the total (increased) amount of IL-4 captured in untreated wt and B6.TCR-Vγ4/6KO mice was almost the same (~ 400 pg/ml serum) (Fig 2). Importantly, a large decrease of captured IL-4 in the mice pre-treated with anti TCR-β mAbs, but not in those treated with the other mAbs, revealed that the serum IL-4 in the non-immunized mice is mainly αβ T cell-derived. This bias was similar for both wt and B6.TCR-Vγ4/6KO mice (Fig 2).

We previously showed that marginal zone and follicular B cells are deficient in B6.TCR-Vγ4/6KO mice, and found that removing IL-4 (B6.TCR-Vγ4/6KO/IL-4KO) restored the numbers of these B cells [23]. Consistently, we show here that removing IL-4 abrogated their splenic lymphopenia (Fig 1A). Importantly, the same manipulation also restored splenic CD4+ and CD8+ αβ T cell populations, albeit with much variation between individual mice (Fig 1B and 1C).

In all strains, individual mice varied in their lymphocyte numbers. In part, this might have been due to residual genetic heterogeneity in the backcrossed mice (see Methods), or age-related, as mice were still growing at the included ages of 8–12 weeks. Female mice consistently had lower body weights (S5 Fig), but we included mice of both genders in the groups because splenic lymphocyte numbers (S6 Fig) and effects of γδ-deficiencies on splenic αβ T cells (S7 Fig) were indistinguishable.

### γδ T cells shape memory-phenotype CD8+ αβ T cell populations in non-immunized mice

Mice harbor substantial numbers of memory-phenotype (MP) CD8+ αβ T cells, even if they have not been exposed to foreign antigens. These cells are distinguished by their high level of expression of CD122 and CD44 [33]. Homeostatic expansion under lymphopenic conditions, and certain cytokines including IL-7, IL-4 and IL-15, can further increase this population [34–36]. Like naïve αβ T cells, MP cells have a diverse TCR repertoire, but they respond to inflammation and infections more rapidly and strongly, and provide better host protection during primary immune responses [37].

**Fig 3:**
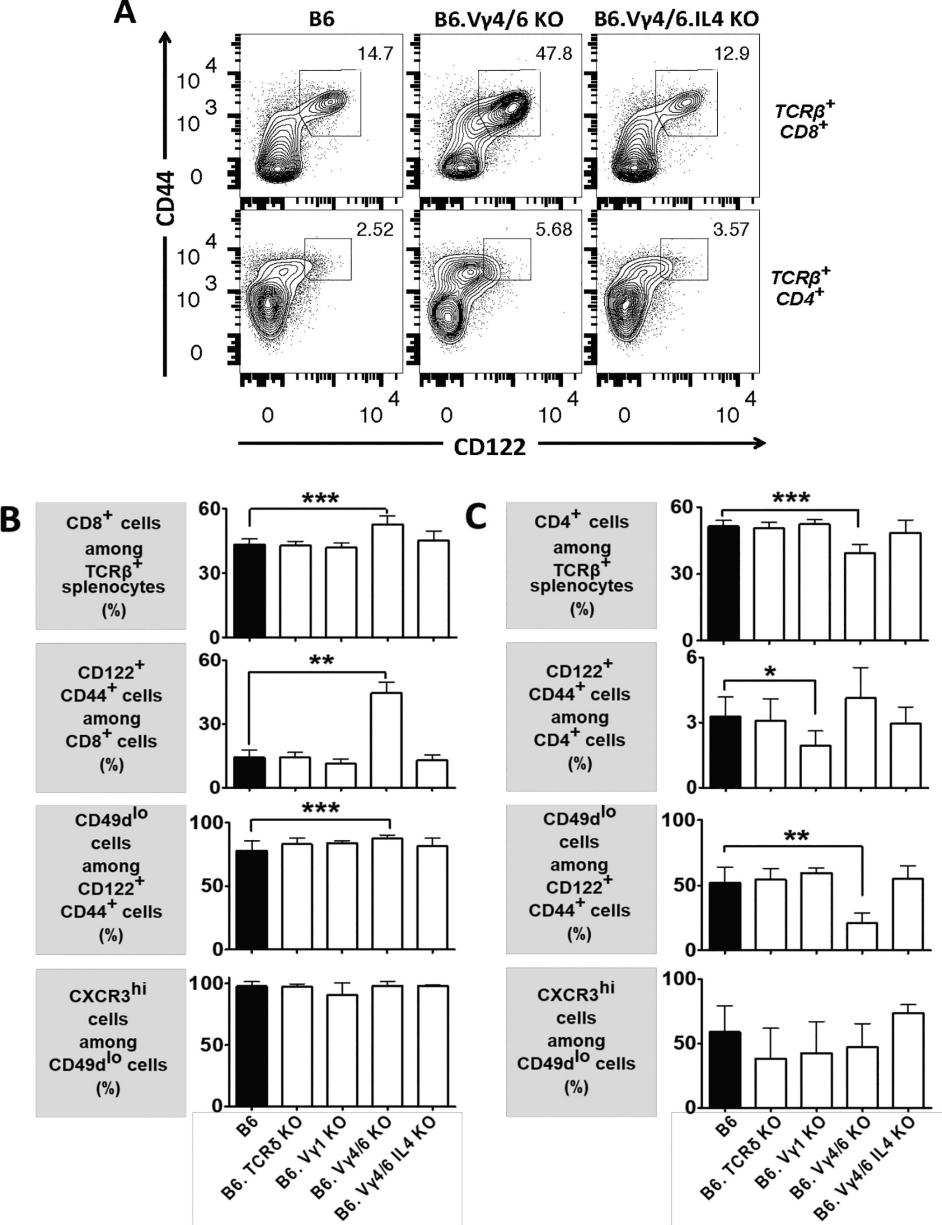
Changes of peripheral memory phenotype αβ T cell populations in γδ T cell-deficient mice. A) Comparison of splenic CD8+ or CD4+ memory-phenotype αβ T cells in C57BL/6 (wt), B6.TCR-Vγ4/6KO and B6.TCR-Vγ4/6KO/IL-4KO mice. The representative flow cytograms shows a >3fold relative increase in the larger population of CD8+ memory-phenotype αβ T cells in a B6.TCR-Vγ4/6KO mouse. CD4+ αβ T cells expressing CD44 at high levels were also increased but only few of these expressed CD122 at high levels. Removal of IL-4 (B6.TCR-Vγ4/6KO/IL-4KO) restored near normal (wt) frequencies of all populations. B,C) Hierarchical comparison of splenic αβ T cells in wt and γδ T cell deficient mice (same strains as in Fig 1), including first CD8+ or CD4+ cells among TCR-β+ T cells, second CD122+CD44+ cells among either type of single-positive αβ T cells, third CD49d low cells among the memory phenotype single-positive αβ T cells, and fourth CXCR3 high cells among CD49d low memory phenotype single-positive αβ T cells. The hierarchical comparison is extended further in Table 1. Female and male mice ages 8–12 wks were included in the comparisons of Fig 2B. n equal or greater than 9 mice/group. *p<0.05, **p<0.01, ***p<0.001.

Having found lymphopenia and higher-than wt IL-4 serum levels and production by T cells in certain γδ T cell-deficient mouse strains [18, 23] (and current study), we examined αβ T cells in the spleen focusing on MP cells (Fig 3) (gating strategy in S8 Fig). Representative cytograms (Fig 3A, top row) show that the percentage of CD8+ αβ T cells with a memory phenotype was much increased in B6.TCR-Vγ4/6KO mice, but not in B6.TCR-Vγ4/6KO/IL-4KO mice. Consistently [38], a subset of the CD8+ αβ T cells in B6.TCR-Vγ4/6KO mice, but not in B6.TCR-Vγ4/6KO/IL-4KO mice, expressed IL-4Rα at increased levels (S9 Fig). Because overall numbers of CD8+ αβ T cells in the spleens of wt and B6.TCR-Vγ4/6KO mice did not significantly differ (Fig 1), the increased frequency of MP CD8+ αβ T cells in B6.TCR-Vγ4/6KO mice reflects a selective expansion of these cells. In contrast to B6.TCR-Vγ4/6KO mice, no substantial changes of MP CD8+ αβ T cells were seen in B6.TCRδKO or B6.TCR-Vγ1KO mice (S10 Fig). Fig 3B shows a comparison of all mouse strains of this study with regard to the frequencies of splenic CD8+ αβ T cells and MP subsets. In B6.TCR-Vγ4/6KO mice but not in the other mice, CD8+ αβ T cells were increased, with a predominance of CD122+CD44+ MP cells. B6.TCR-Vγ4/6KO/IL-4KO mice had normal frequencies (and numbers) of such cells. As with wt mice and the other mouse strains, almost all of the expanded MP CD8+ αβ T cells in B6.TCR-Vγ4/6KO mice expressed CD49d at low levels. This is typical for antigen-inexperienced (AI) MP αβ T cells (which would not, presumably, have been previously stimulated via their TCRs), because CD49d is up-regulated following stimulation via the TCR [33]. Therefore, the data suggest that the Vγ4+ and/or Vγ6+ γδ T cells present in wt mice function to control the size of the population of AIMP CD8+ αβ T cells and that IL-4 plays a role in this regulation.

AIMP CD8+ αβ T cells that arise as a result of lymphopenia-induced peripheral homeostatic proliferation [34], “virtual memory” (VM) MP T cells in the periphery, and IL-4-dependent innate MP αβ T cells in thymus and periphery, all differ in their gene expression [34, 37]. In order to determine which might be affected by γδ T cells, we examined products of such differentially expressed genes including CXCR3, a CXC chemokine receptor involved in sensing and responding to inflammation, and the transcription factors eomesodermin (EOMES) and T-bet. Nearly all of the CD122+CD44+CD49dlo AIMP CD8+ splenic αβ T cells detected in the mouse strains of this study highly expressed CXCR3 (Fig 3B), and among these CXCR3high cells, almost all expressed T-bet and EOMES (Tables 1 and 2). The nearly uniform transcription factor expression pattern among the expanded CD8+ MP αβ T cells in B6.TCR-Vγ4/6KO mice resembles that of the previously described VM CD8+ αβ T cells [34, 37, 39, 40].

**Table 1:** T-bet and EOMES expression among antigen-inexperienced memory-phenotype αβ T cells, cell frequencies.

	%	B6	B6.Vγ4/6KO	B6.Vγ4/6KO/ IL-4KO
**TCRβ**^**+**^ **CD8**^**+**^ **CD122**^**+**^**CD44**^**+**^ **CD49d**^**lo**^ **CXCR3** ^**hi**^	**T-bet**^**+**^ **EOMES**^**+**^	97.4 ± 0.3	98.4 ± 0.5	95.1 ± 0.5
	**T-bet**^**-**^ **EOMES**^**+**^	0.1 ± 0.0	0.0 ± 0.0	0.1 ± 0.0
	**T-bet**^**-**^ **EOMES**^**-**^	0.1 ± 0.0	0.0 ± 0.0	0.1 ± 0.1
**TCRβ**^**+**^ **CD4**^**+**^ **CD122**^**+**^**CD44**^**+**^ **CD49d**^**lo**^ **CXCR3** ^**hi**^	**T-bet**^**+**^ **EOMES**^**-**^	98.6 ± 0.3	85.7 ± 3.8	95.5 ± 1.6
	**T-bet**^**+**^ **EOMES**^**+**^	0.9 ± 0.3	13.9 ± 3.9	4.0 ± 1.6
	**T-bet**^**-**^ **EOMES**^**+**^	0.0 ± 0.0	0.4 ± 0.4	0.0 ± 0.0
	**T-bet**^**-**^ **EOMES**^**-**^	0.4 ± 0.2	0.0 ± 0.0	0.5 ± 0.2

Data are presented as means +/- SD; groups were compared using the nonparametric ANOVA (Kruskal Wallis) test. C57BL/6 (B6) mice (n = 8), B6. Vγ4/6KO mice (n = 3), and B6. Vγ4/6KO/IL-4KO mice (n = 7)

**Table 2:** T-bet and EOMES expression among antigen-inexperienced memory-phenotype αβ T cells, cell numbers.

	numbers (x 10^4^)	B6	B6.Vγ4/6KO	B6.Vγ4/6KO/ IL-4KO
**TCRβ**^**+**^ **CD8**^**+**^ **CD122**^**+**^**CD44**^**+**^ **CD49d**^**lo**^ **CXCR3** ^**hi**^	**Tbet**^**+**^ **EOMES**^**-**^	3.1 ± 0.3	5.0 ± 2.2	10.9 ± 3.9
	**T-bet**^**+**^ **EOMES**^**+**^	138.4 ± 17.6	314.5 ± 79.2	228.8 ± 88.5
	**T-bet**^**-**^ **EOMES**^**+**^	0.2 ± 0.1	0.1 ± 0.1	0.3 ± 0.1
	**T-bet**^**-**^ **EOMES**^**-**^	0.2 ± 0.0	0.0 ± 0.0	0.2 ± 0.1
**TCRβ**^**+**^ **CD4**^**+**^ **CD122**^**+**^**CD44**^**+**^ **CD49d**^**lo**^ **CXCR3** ^**hi**^	**T-bet**^**+**^ **EOMES**^**-**^	23.5 ± 2.8	3.7 ± 0.9	25.3 ± 6.0
	**T-bet**^**+**^ **EOMES**^**+**^	0.4 ± 0.1	0.5 ± 0.0	1.1 ± 0.6
	**T-bet**^**-**^ **EOMES**^**+**^	0.0 ± 0.0	0.0 ± 0.0	0.0 ± 0.0
	**T-bet**^**-**^ **EOMES**^**-**^	0.1 ± 0.1	0.0 ± 0.0	0.2 ± 0.1

Data are presented as means +/- SD; groups were compared using the nonparametric ANOVA (Kruskal Wallis) test. C57BL/6 (B6) mice (n = 8), B6. Vγ4/6KO mice (n = 3), and B6. Vγ4/6KO/IL-4KO mice (n = 7)

### γδ T cells in non-immunized mice also affect memory-phenotype CD4+ αβ T cells

Whereas CD8+ MP αβ T cells in non-immunized mice have been examined extensively [34, 37, 41], not much attention has been given to the less frequent CD4+ MP αβ T cells [42, 43]. In the wt mice of this study, only 2–4% of CD4+ splenic αβ T cells fell into this category (Fig 3A, bottom row, Fig 3C) compared to 13–15% of CD8+ splenic αβ T cells (Fig 3A, top row, Fig 3B). In B6.TCRδKO mice (Fig 3C, S10 Fig), the frequency of MP cells among CD4+ αβ T cells was similarly low. In most but not all B6.TCR-Vγ1KO mice (Fig 3C, S10 Fig), MP cells among CD4+ αβ T cells were even further diminished. In some B6.TCR-Vγ4/6KO mice, on the other hand, MP cells seemed to be slightly increased (Fig 3A, bottom row), but overall, these mice did not significantly differ from wt mice (Fig 3C). Whereas nearly all MP CD8+ αβ T cells in the mouse strains examined were CD49dlow (Fig 3B), only about one half of MP CD4+ αβ T cells displayed this “antigen-inexperienced” phenotype in wt mice, and in B6.TCRδKO and B6.TCR-Vγ1KO mice (Fig 3C). The other MP CD4+ αβ T cells expressed CD49d at high levels, perhaps reflecting better chances of MP CD4+ αβ T cells in non-immunized mice (compared to MP CD8+ αβ T cells) to experience TCR-stimulation. Quite distinctly, even fewer of these cells (~20%) were CD49d low in B6.TCR-Vγ4/6KO mice (Fig 3C). In contrast, B6.TCR-Vγ4/6KO/IL-4KO mice resembled wt mice, suggesting that (when compared to MP CD8+ αβ T cells) IL-4 might have the opposite effect on MP CD4+ αβ T cells in non-immunized mice, and actually favor antigen-experienced cells in this population. Only about one half of the few MP CD4+ αβ T cells that did express CD49d at low levels expressed CXCR3 highly, and most of these cells were T-bet+ EOMES-, as opposed to CXCR3hi CD49dlo MP CD8+ αβ T cells, which were mostly T-bet+ EOMES+ (Tables 1 and 2).

### The influence of γδ T cells on developing αβ T cells becomes evident late during intra-thymic maturation

**Fig 4:**
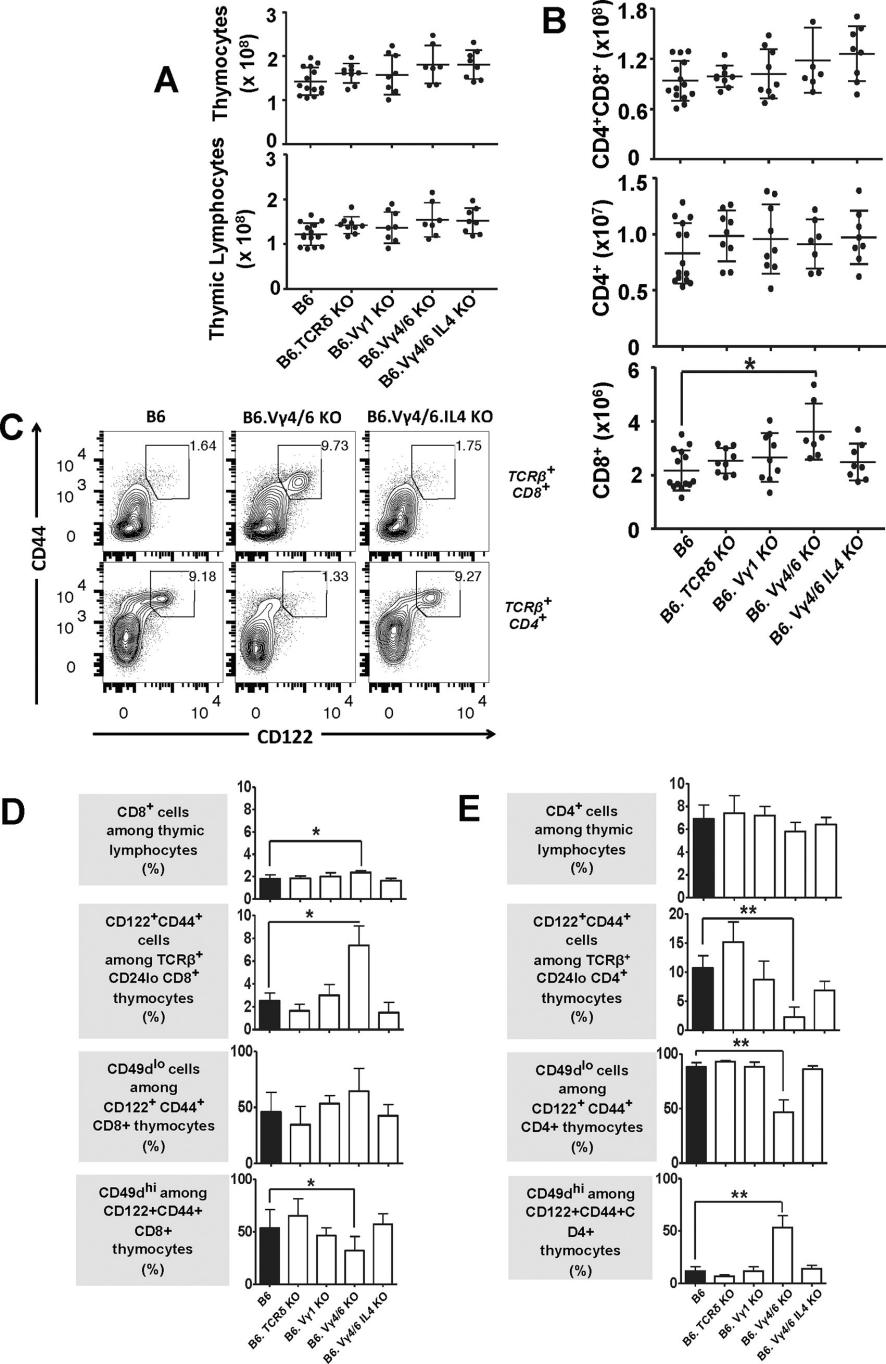
Thymocytes in γδ T cell-deficient mice. A) No lymphopenia in the thymus of in γδ T cell-deficient mice. Total thymocytes and thymic lymphocytes (identified by their scatter profile) were counted in the mouse strains of this study (same strains as in Fig 1). Male and female mice, ages 8–12 wks were included. n equal or greater than 7 mice/group. B) No deficiency of double- or single-positive thymocytes in γδ T cell-deficient mice. Double (CD4+CD8+) and single (CD4+ or CD8+) positive thymic lymphocytes were counted in the mouse strains of this study (same strains as in Fig 1). Male and female mice, ages 8–12 wks were included. n equal or greater than 7 mice/group. *p<0.05. C) Comparison of CD8+ or CD4+ single-positive CD122+CD44+ TCR-β+ thymocytes in C57BL/6 (wt), B6.TCR-Vγ4/6KO and B6.TCR-Vγ4/6KO/IL-4KO mice. The representative flow cytograms show an increase in the relative frequency of CD8-single-positive CD122+CD44+ TCR-β+ thymocytes in B6.TCR-Vγ4/6KO mice, and a relative decrease of CD4-single-positive CD122+CD44+ TCR-β+ thymocytes. Both cell-types were present at normal (wt) frequencies in B6.TCR-Vγ4/6KO/IL-4KO mice. D, E) Hierarchical comparison of mature TCR-β+ thymocytes in wt and γδ T cell deficient mice (same strains as in Fig 1), including first CD8- or CD4-single-positive cells among thymic lymphocytes, second CD122+CD44+ cells among either type of single-positive αβ T thymocytes, third CD49d low cells among the memory phenotype single-positive αβ thymocytes and fourth, CD49d high cells among memory phenotype single-positive αβ thymocytes. Female and male mice ages 8–12 wks were included in the comparisons of Fig 1A. n equal or greater than 7 mice/group. *p<0.05, **p<0.01.

In contrast to the spleen, there was no indication of lymphopenia in the thymi of γδ T cell-deficient mouse strains (Fig 4, gating strategy in S11 Fig). Numbers of non-gated thymocytes and of total thymic lymphocytes (Fig 4A) were mostly unchanged or perhaps slightly higher compared to those in wt mice. We did not analyze the earliest precursors of αβ T cells (CD4/CD8 double-negative thymocytes), but numbers of their immediate progeny, double-positive “immature” thymocytes were essentially unchanged. Likewise, CD4-single-positive CD24low “mature” thymic lymphocytes (Fig 4B) were unchanged, and only CD8 single-positive “mature” thymic lymphocytes in B6.TCR-Vγ4/6KO mice were significantly increased relative to those in wt mice. The numeric changes in “mature” CD8 single-positive thymocytes increased the relative frequency of these cells and slightly decreased the frequency of CD4+ thymocytes (S12 Fig). Similarly to CD8+ αβ T cells in the spleen, a much-increased fraction of these expanded CD8 single-positive mature thymocytes in B6.TCR-Vγ4/6KO but not in B6.TCR-Vγ4/6KO/IL-4KO mice expressed the activation/memory markers CD122 and CD44 (representative cytograms in Fig 4C, top row, comparison with other mouse strains Fig 4D). Among the mouse strains tested, only the CD8+CD122+CD44+ thymocyte population in B6.TCR-Vγ4/6KO mice contained CD49d high cells at a lower frequency when compared to the wt mice, but the difference was small (Fig 4D). These data appear to reflect a limited role for IL-4 in the expansion of MP CD8-single positive thymic lymphocytes, and might indicate that AIMP CD8+ αβ T cells originate in the thymus (at least in B6.TCR-Vγ4/6KO mice).

In stark contrast to the increased MP CD8+ mature thymic lymphocytes, MP CD4+ mature thymic lymphocytes were much decreased in B6.TCR-Vγ4/6KO mice when compared to wt mice (Fig 4C, bottom row, Fig 4E), a difference we had not observed with αβ T cells in the spleen of B6.TCR-Vγ4/6KO mice (Fig 3C). Among these thymic lymphocytes, CD49d expression was skewed towards high levels when compared to the other mouse strains (consistent with stimulation via the TCR) (Fig 4E), far more prominently so than the pattern observed in the spleen (Fig 3). B6.TCR-Vγ4/6KO/IL-4KO mice had normal (low) frequencies of CD49d high cells among their MP CD4+ thymic lymphocytes, again linking the expression pattern to IL-4.

### γδ T cells appear to impact αβ T cells and B cells at similar stages in their development

**Fig 5:**
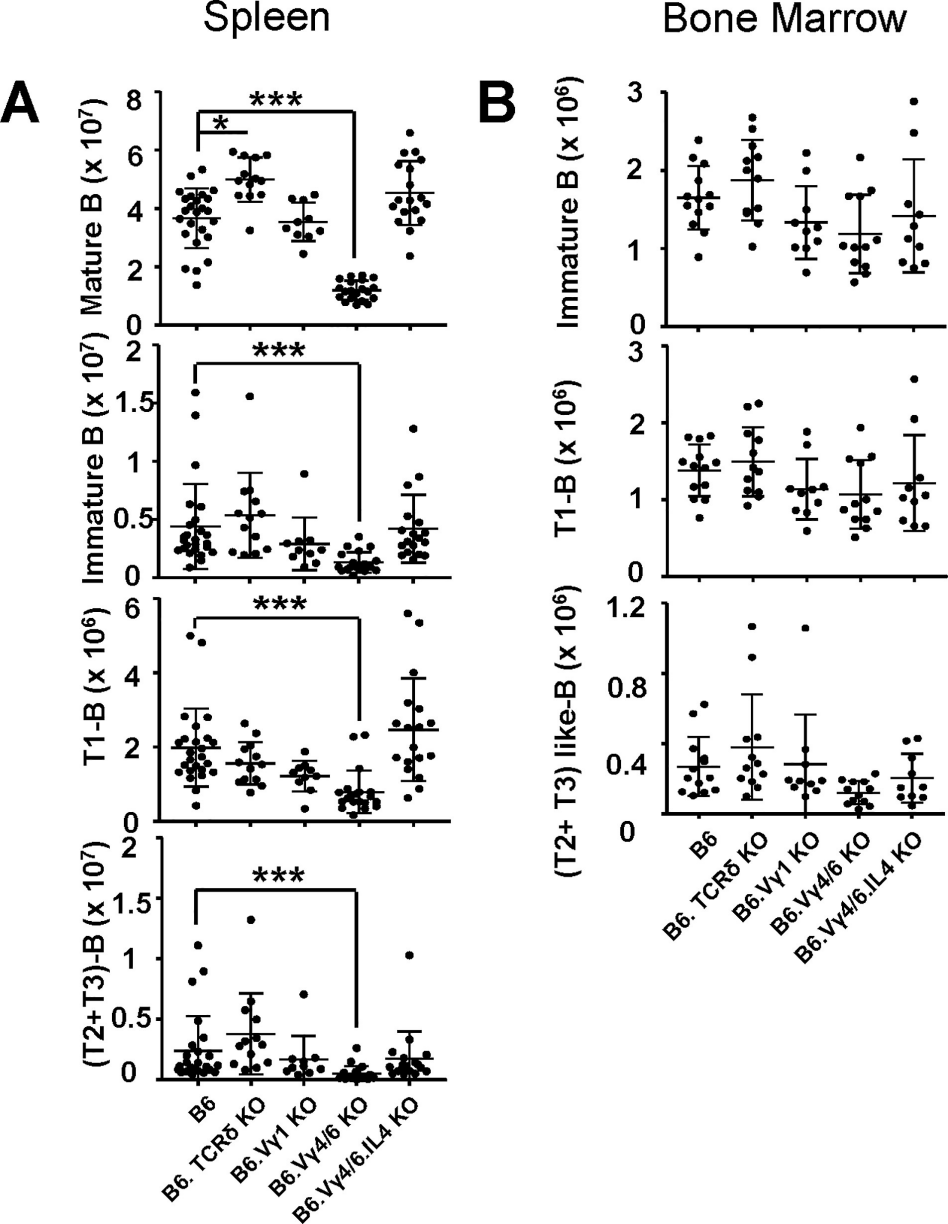
Changes of transitional B cells in spleen and transitional-like B cells in bone marrow of γδ T cell-deficient mice. A) Comparison of splenic B cells (total number of cells/spleen) including mature (B220+IgM+CD93-), immature (B220+IgM+CD93+), transitional 1 (T1, B220+IgM+CD93+CD23-) and transitional 2+3 (T2+T3, B220+IgM+CD93+CD23+) B cells, same mouse strains as in Fig 1. B) Comparison of bone marrow B cells (total number of B cells/right femur) including immature (B220+IgM+CD93+), transitional 1-like (T1, B220+IgM+CD93+CD23-) and transitional 2+3-like (T2+T3, B220+IgM+CD93+CD23+) B cells, same mouse strains as in Fig 1. Female and male mice were included in the comparisons shown in Fig 5, ages 8–13 wks, n equal or greater 8 mice/group. *p<0.05, ***p<0.001.

We had previously seen large differences of mature and immature transitional B cell populations in the spleens of non-immunized γδ T cell-deficient mice (same strains as in the current study) but not of immature B cells represented by Hardy fractions [44] in bone marrow [23]. To ascertain the stage in B cell development at which γδ T cells engage with B cells, we compared transitional B cells in the spleen of these mice with the small transitional-like bone marrow B cell population [45], which marks B cells about to exit from bone marrow (Fig 5). As noted before [23], numbers of mature (B220+IgM+CD93-) splenic B cells were much decreased in B6.TCR-Vγ4/6KO mice (Fig 5A). Because mature B cells in the spleen are more frequent than T cells, their loss accounts for most of the splenic lymphopenia in these mice. Total immature (B220+IgM+CD93+) splenic B cells were also decreased in these mice, and so were early (T1, B220+IgM+CD93+CD23-) and late (T2+T3, B220+IgM+CD93+CD23+) transitional B cells. B6.TCR-Vγ4/6KO/IL-4KO mice showed none of the changes in B cells seen with B6.TCR-Vγ4/6KO mice. By contrast, in bone marrow of B6.TCR-Vγ4/6KO mice, we failed to detect significant losses of immature B cells and their transitional-like subtypes (Fig 5B).

**Fig 6:**
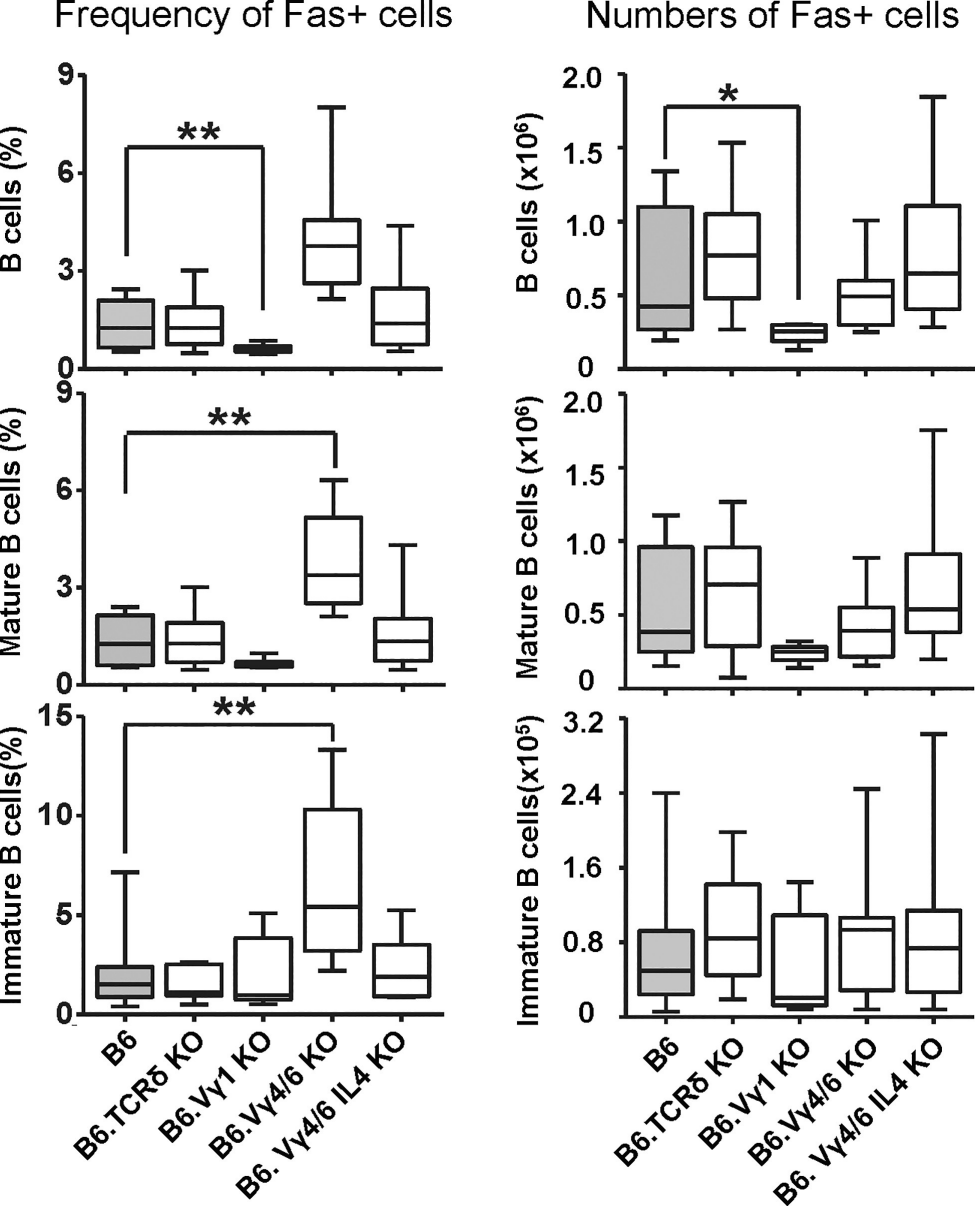
Changes in Fas+ B cells in the spleen of γδ T cell-deficient mice. Comparison of Fas-expressing B cells in γδ T cell-deficient mouse strains for relative frequencies and absolute numbers (same strains as in Fig 1). B cells (B220+IgM+), mature B cells (B220+IgM+CD93-), immature B cells (B220+IgM+CD93+). n equal or greater than 9 mice/group. *p<0.05, **p<0.01.

Expression of the death receptor Fas can serve as a marker of activated/memory B cells [46]. We previously reported increased Fas+ germinal center B cells in the spleens of B6.TCR-Vγ4/6KO mice [18]. Here, we broadly assessed Fas receptor expression by B cells in the spleen, in order to compare frequencies of activated/memory B cells in the non-immunized γδ T cell-deficient mice of this study (Fig 6). Similarly to the MP CD8+ αβ T cells, Fas+ immature and mature B cells of B6.TCR-Vγ4/6KO but not B6.TCR-Vγ4/6KO/IL-4KO mice were increased in frequency although—unlike the T cells, absolute numbers of these B cells were not increased. Inversely, B6.TCR-Vγ1KO mice seemed to suffer an absolute loss of (mostly mature) Fas+ B cells. A comparison of these data with those on αβ T cells suggests that with either lymphocyte-type, the γδ-influence becomes manifest late during pre-immune maturation.

## Discussion

The possibility that γδ T cells affect the immune system prior to its responses has hardly been explored. To address this question, we compared immune cells in a set of genetic background-matched γδ T cell-deficient mouse strains. Between these mouse strains, without immunization, we found differences in overall leucocyte numbers, including granulocytes and B cells [18, 23]. We also observed large differences in antibody levels [18], suggesting that γδ T cells influence the production of natural antibodies [47], and more broadly influence the shape of the native immune system [48]. The current study extends this investigation to αβ T cells. The results suggest that γδ T cells begin to influence the development of αβ T cells late during their intra-thymic maturation, thus shaping mature T cell populations. This pre-immune interaction was most noticeable with memory phenotype (MP) αβ T cells, which might be important because MP αβ T cells—even if they are not specific for relevant antigens—reportedly contribute more to host protection than do naïve T cells [34, 37].

Several shared features connect the current study of αβ T cells in non-immunized mice to the previous work on B cells [18, 23]. First, similarly to the findings with B cells and antibodies, complete absence of γδ T cells seemed to have little effect on αβ T cells in non-immunized mice, whereas selective deficiencies of γδ T cell-subsets were associated with large changes in αβ T cells. This particular feature seems to reflect some interactive balance between functionally differentiated γδ T cell-populations [18, 48–50]. Perturbation of the balance drastically changes the influence of the remaining γδ T cells on other cell-types. Interestingly, the balance seems to be maintained in part through internal crosstalk between γδ T cells. We believe this to be true because we previously observed that loss of certain γδ T cell-types (in B6.TCR-Vγ4/6KO mice) changed population size and functional activity of the remaining γδ T cells (i.e. Vγ1+ and Vγ7+ cell populations in B6.TCR-Vγ4/6KO mice) [18, 48]. Second, and again similarly to the findings with B cells, γδ T cell-regulated IL-4 seems to be a critical mediator of the γδ-effect on pre-immune αβ T cells in the γδ T cell-deficient mice. In earlier studies, we found increased numbers of IL-4-producing T cells (both αβ and γδ T cells) and elevated levels of circulating IL-4 in B6.TCR-Vγ4/6KO mice [18, 23], along with numerous cellular and molecular changes expected to occur with increased IL-4 activity [51–54]. These changes were not present in B6.TCR-Vγ4/6KO/IL-4KO mice. Likewise, in the current study, non-immunized B6.TCR-Vγ4/6KO but not B6.TCR-Vγ4/6KO/IL-4KO mice harbored enlarged populations of memory-phenotype (MP) CD8+ αβ T cells, some of which are known to depend on IL-4 [34, 37]. In this study, we confirmed that most of the elevated IL-4 in B6.TCR-Vγ4/6KO is T cell-derived. Moreover, in both non-immunized B6.TCR-Vγ4/6KO mice and C57BL/6 (wt) mice, αβ T cells appear to be the major source of the circulating IL-4. It remains to be determined exactly how certain γδ T cells control pre-immune IL-4-production by other cell-types. In any case, the effect of γδ T cell-controlled IL-4 on B and T cells is mostly evident with their activated/memory states. Thus, in B6.TCR-Vγ4/6KO mice but not in B6.TCR-Vγ4/6KO/IL-4KO mice, we found both immature and mature, activated Fas+ B cells [46] to be much increased in frequency (though not in numbers) in the presence of huge losses of resting B cells. Likewise, CD122+CD44+ MP αβ T cells were increased (in frequency and numbers) while other αβ T cells were unchanged or lost. Secondly, the data of this study suggest that γδ T cells begin to exert their influence on αβ T cells already in the thymus, at a late single-positive (medullary?) stage of intra-thymic maturation. Of note, we cannot exclude an influence of γδ T cells on DN thymocyte precursors of αβ T cells but this seems unlikely given that we did not find significant changes in DP thymocytes. Again, this is reminiscent of their influence on B cells, which becomes evident at the transitional stages of B cell-development [23] (and this study). Similarly to the effects on developing B cells, the γδ-dependent changes of developing CD8+ MP thymocytes correlate well with changes of mature CD8+ MP αβ T cells.

The parallels between changes of B cells and αβ T cells in the γδ T cell-deficient mice suggest that similar mechanisms are at work. This is informative because unlike B cells, αβ T cells can express TCR-γ and, sometimes, –δ genes, and thus potentially could be intrinsically affected by the mutations of the γδ T cell-deficient mouse strains examined here. However, with B cells, non-genetic manipulations of γδ T cells supported the notion of cellular interactions between γδ T cells and B cells as cause for the changes in B cells and antibodies [18, 22, 23, 25]. On the basis of the findings with B cells, and because of the similarity of the γδ-effects on B and αβ T cells, we would expect that cellular interactions (rather than intrinsic effects of the mutations) also mediate the changes in αβ T cells.

Consistently, the data imply that the γδ-influence on B cells and αβ T cells is mainly mediated by IL-4. IL-4 directly supports T and B cell-development, during the immune responses and before [51, 55]. Early studies with fetal thymocytes characterized IL-4 as a growth and differentiation factor for intra-thymic T cell precursors [56, 57], but inhibitory effects of IL-4 on thymocyte development were seen as well [58]. In the first IL-4 transgenic mice, overexpression of IL-4 lead to lymphopenia and death, so that transgenic IL-4 expression had to be attenuated in order to establish transgenic mouse strains [59]. Direct cytotoxicity and IL-4-induced apoptosis has been described with human B-lineage acute lymphoblastic leukemia cells as well as with a proportion of normal B cell progenitors [60]. In addition, we observed increased IL-21 production in B6.TCR-Vγ4/6KO mice but not in B6.TCR-Vγ4/6KO/IL-4KO mice, linking IL-4 and IL-21 (Kira Rubtsova, unpublished data). IL-21 can mediate growth arrest and apoptosis of certain B cells [61, 62]. Regarding T cells, a cytokine receptor consisting of IL-4Rα and IL-13Rα1 (heteroreceptor, HR) was found to transduce IL-4 (and IL-13) signals that drive apoptosis of Th1 cells and skew neonatal immunity towards Th2 cells [63], and earlier in development, IL-4/IL-13 signaling through the HR can affect the potential of thymic progenitors to commit to the T cell lineage [64]. Therefore, it is conceivable that γδ T cells, through their control of IL-4, promote the loss of (naïve?) B and T lymphocytes, while simultaneously protecting and expanding activated B and T cell populations.

Lastly, our data indicate that γδ T cells affect CD4+ and CD8+ MP αβ T cells differently. The expansion of CD8+ MP αβ T cells in B6.TCR-Vγ4/6KO mice suggests that γδ T cells deficient in these mice normally repress CD8+ MP αβ T cells, in thymus and spleen. The repression could be direct or via a second, otherwise supportive cell-type, e.g. Vγ1+ γδ T cells. The expanded CD8+ αβ T cells in B6.TCR-Vγ4/6KO mice closely resemble antigen-inexperienced memory-phenotype (AIMP) CD8+ αβ T cells previously described by others [34, 37]. In the same mice, CD4+ (CD49d low and high) MP αβ T cells were diminished, albeit only in the thymus. Here, the developing αβ T cells might be normally supported by the γδ T cells deficient in B6.TCR-Vγ4/6KO mice, or suppressed by some of those still present. Specifically, Vγ1+ γδ T cells, when freed from the inhibition by Vγ4+ and/or Vγ6+ γδ T cells, might prevent the development of CD4+ MP αβ T cells. In apparent contradiction, a contraction of CD4+ MP αβ T cells in the spleen (but not in the thymus) of B6.TCR-Vγ1KO mice suggests that Vγ1+ γδ T cells normally support CD4+ MP αβ T cells, at least in the periphery. A resolution of this apparent contradiction might lie in our earlier observation of functional differences between Vγ1+ γδ T cells developing in different environments [18, 23]. Those developing in B6.TCR-Vγ4/6KO mice are type 2-biased, hence better equipped to support IL-4-dependent CD8+ AIMP αβ T cells and perhaps more likely to inhibit type-1 IL-4-independent CD4+ MP αβ T cells. In contrast, Vγ1+ γδ T cells in wt mice, developing in the presence of all other γδ T cells, tend to be type 1-biased, and thus may support primarily type-1 IL-4-independent CD4+ MP αβ T cells. Further studies will be required to fully assess and delineate the influence of γδ T cells on pre-immune αβ T cells and B cells.

## Supporting information

S1 Fig Effect of *in vivo* treatment with anti TCR mAbs on splenic T cells in C57BL/6 (wt) mice. Adult mice were treated with i.v. injected anti TCR mAbs or saline alone, and analyzed by flow cytometry as detailed in the Methods.
(PDF)

S2 Fig Effect of in vivo treatment with anti TCR mAbs on splenic T cells in B6.TCR-Vγ4/6 KO mice. Adult mice were treated with i.v. injected anti TCR mAbs or saline alone, and analyzed by flow cytometry as detailed in the Methods.
(PDF)

S3 Fig No significant effect of in vivo treatment with anti TCR mAbs on splenic B cells in C57BL/6 (wt) and B6.TCR-Vγ4/6 KO mice. Adult mice were treated with i.v. injected anti TCR mAbs or saline alone, and analyzed by flow cytometry as detailed in the Methods.
(PDF)

S4 Fig Relative frequencies of CD4+ and CD8+ αβ T cells in the spleen of γδ T cell-deficient mice. Female and male mice ages 8–12 wks were included in the comparisons shown (same mice as in Fig 1). ***p<0.001.
(PDF)

S5 Fig Growth of C57BL/6 mice and background-matched γδ T cell deficient mice. A) Comparison of live body weight (grams) of female C57BL/6 (B6), B6.TCRδKO, B6.TCR-Vγ1KO, B6.TCR-Vγ4/6KO, and B6.TCR-Vγ4/6KO/IL-4KO mice, at 4, 8 and 12 wks of age.
B) Same comparison as in A but with male mice.
n equal or greater 5 mice/group, except for B6.TCR-Vγ1KO mice (females: 8 wks n = 1, 12 wks n = 2, males: 12 wks n = 2) and C57BL/6 mice (males: 12 wks n = 3).
(PDF)

S6 Fig Similar splenic lymphocyte numbers in female and male γδ T cell deficient mice. Comparison of age-matched female and male mice for splenic lymphocyte numbers, including C57BL/6 (B6), B6.TCRδKO, B6.TCR-Vγ1KO, B6.TCR-Vγ4/6KO, and B6.TCR-Vγ4/6KO/IL-4KO mice. Female and male mice ages 8–12 wks were included in the comparison shown in S5 Fig. n equal or greater than 5 mice/group.
(PDF)

S7 Fig Similar effect of γδ T cell-deficiencies on total splenic αβ T cells in female and male mice. Comparison of total numbers of TCR-β+ cells in the spleen of C57BL/6 (B6), B6.TCRδKO, B6.TCR-Vγ1KO, B6.TCR-Vγ4/6KO, and B6.TCR-Vγ4/6KO/IL-4KO mice. Female and male mice ages 8–12 wks were included in the comparison shown in S6 Fig. n equal or greater than 10 mice/group.
(PDF)

S8 Fig Gating strategy for CD4+ and CD8+ memory-phenotype αβ T cells in the spleen of γδ T cell deficient mice. (PDF)

S9 Fig IL4Rα Expression on CD4 and CD8 T cells. Splenocytes were stained and analyzed by flow cytometry as described in the Methods.
(PDF)

S10 Fig CD4+ and CD8+ memory-phenotype αβ T cells in the spleen of C57BL/6 (wt), B6.TCRδKO and B6.TCR-Vγ1KO mice. Unlike B6.TCR-Vγ4/6KO mice, no substantial changes in the frequencies of memory-phenotype αβ T cells were found in the spleens of B6.TCRδKO and B6.TCR-Vγ1KO mice.
(PDF)

S11 Fig Gating strategy for memory-phenotype CD4 and CD8 single-positive thymocytes. (PDF)

S12 Fig Relative frequencies of CD4+/CD8+, CD4+ and CD8+ thymocytes in γδ T cell-deficient mice. Male and female mice, ages 8–12 wks were included (same mice as in Fig 4). n equal or greater than 7 mice/group. *p<0.05, **p<0.01.
(PDF)
