# Supplementary figures and images for: γδ T cells shape memory-phenotype αβ T cell populations in non-immunized mice
Source: PLoS One. 2019 Jun 25;14(6):e0218827. doi: 10.1371/journal.pone.0218827 (PMC6592556; doi:10.1371/journal.pone.0218827)

**S1 Fig. : Effect of in vivo treatment with anti TCR mAbs on splenic T cells in C57BL/6 (wt) mice**

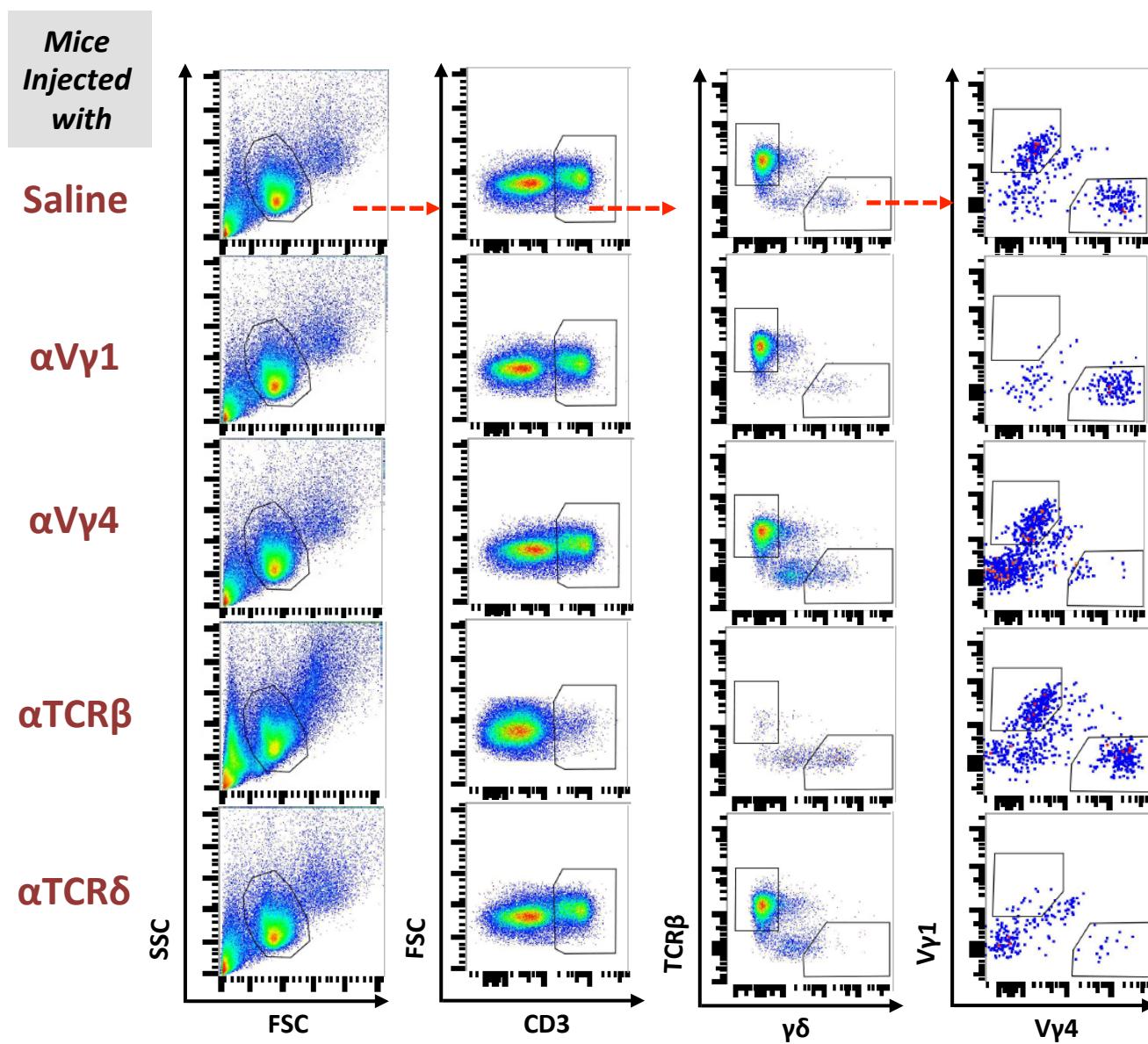

Supplement: S1 Fig — Adult mice were treated with i.v. injected anti TCR mAbs or saline alone, and analyzed by flow cytometry as detailed in the Methods. (PDF) [file pone.0218827.s001.pdf]

**S2 Fig. : Effect of in vivo treatment with anti TCR mAbs on splenic T cells in B6.TCR-V $\gamma$ 4/6 KO mice**

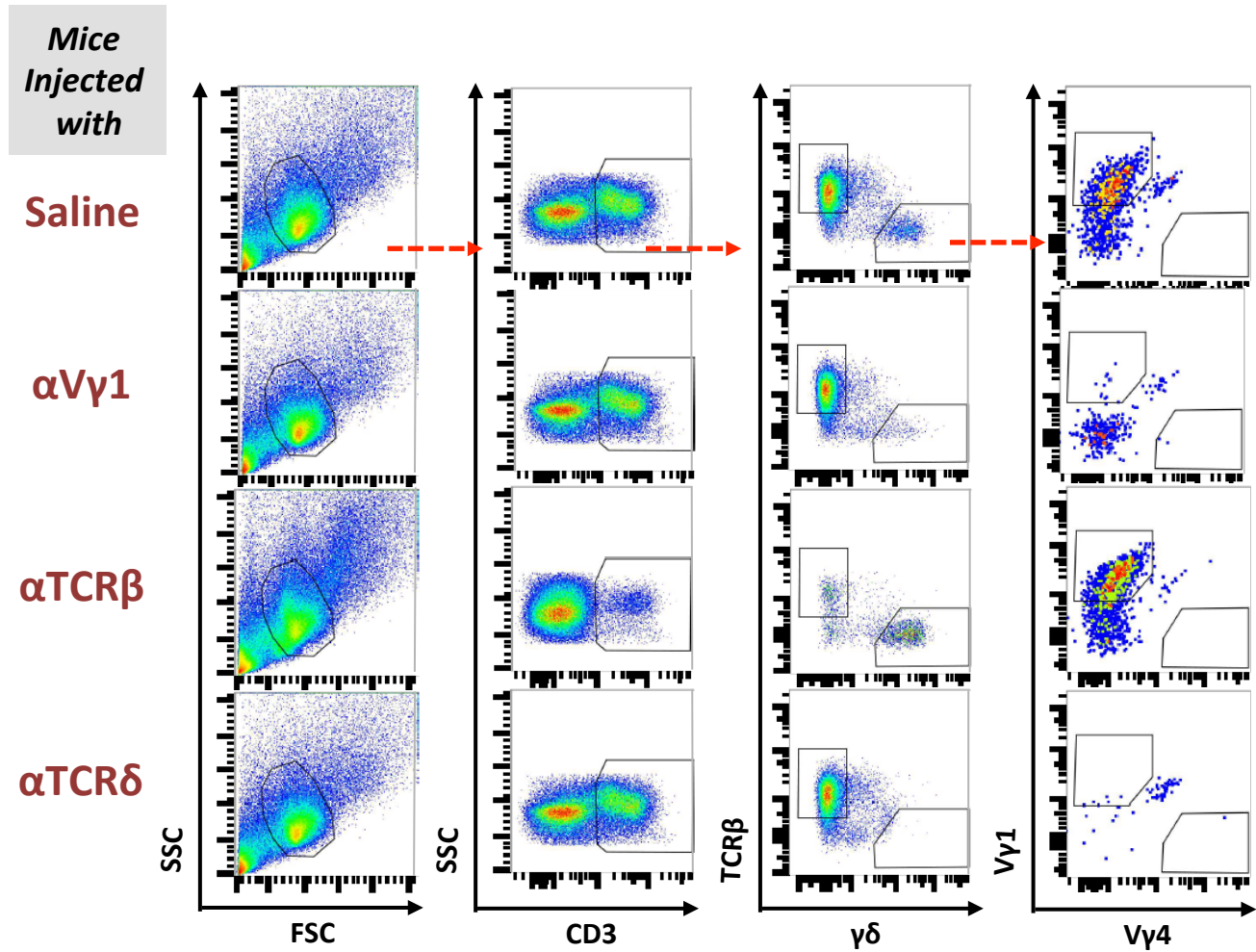

Supplement: S2 Fig — Adult mice were treated with i.v. injected anti TCR mAbs or saline alone, and analyzed by flow cytometry as detailed in the Methods. (PDF) [file pone.0218827.s002.pdf]

S5 Fig.: Growth of C57BL/6 mice and background-matched  $\gamma\delta$  T cell deficient mice

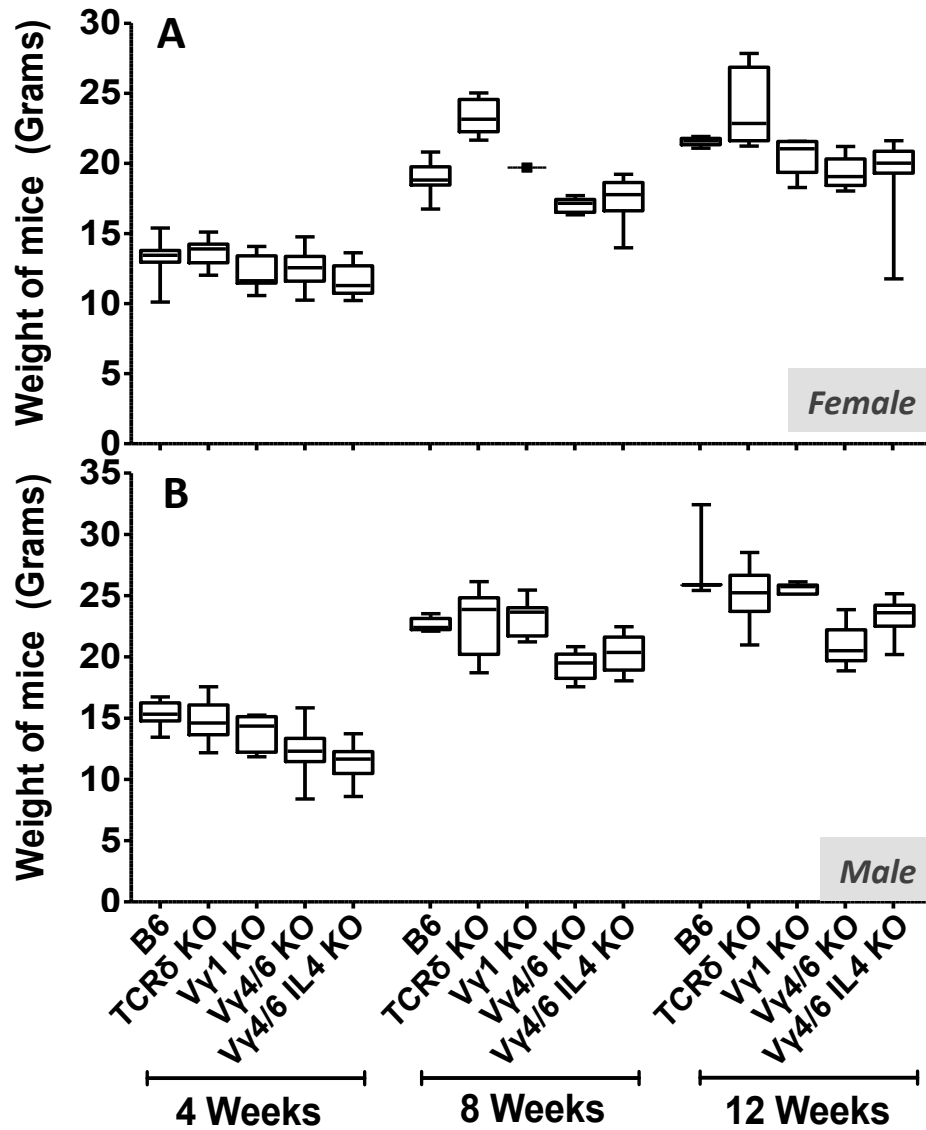

Supplement: S5 Fig — A) Comparison of live body weight (grams) of female C57BL/6 (B6), B6.TCRδKO, B6.TCR-Vγ1KO, B6.TCR-Vγ4/6KO, and B6.TCR-Vγ4/6KO/IL-4KO mice, at 4, 8 and 12 wks of age. B) Same comparison as in A but with male mice. n equal or greater 5 mice/group, except for B6.TCR-Vγ1KO mice (females: 8 wks n = 1, 12 wks n = 2, males: 12 wks n = 2) and C57BL/6 mice (males: 12 wks n = 3). (PDF) [file pone.0218827.s005.pdf]

S6 Fig.: Similar splenic lymphocyte numbers in female and male  $\gamma\delta$  T cell deficient mice

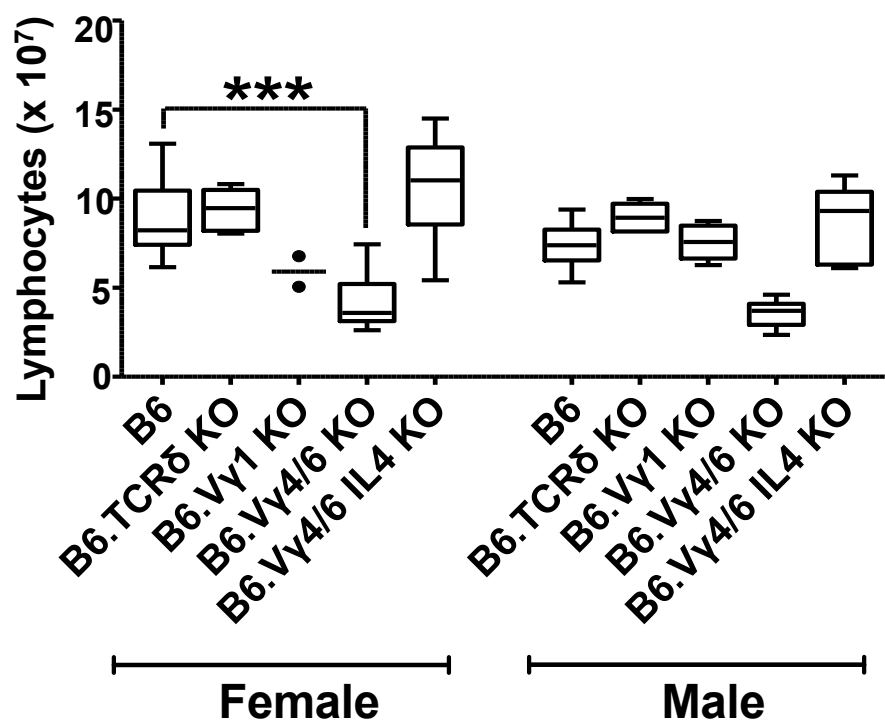

Supplement: S6 Fig — Comparison of age-matched female and male mice for splenic lymphocyte numbers, including C57BL/6 (B6), B6.TCRδKO, B6.TCR-Vγ1KO, B6.TCR-Vγ4/6KO, and B6.TCR-Vγ4/6KO/IL-4KO mice. Female and male mice ages 8–12 wks were included in the comparison shown in S5 Fig. n equal or greater than 5 mice/group. (PDF) [file pone.0218827.s006.pdf]

S9 Fig.: IL4R $\alpha$  Expression on CD4 and CD8 T cells

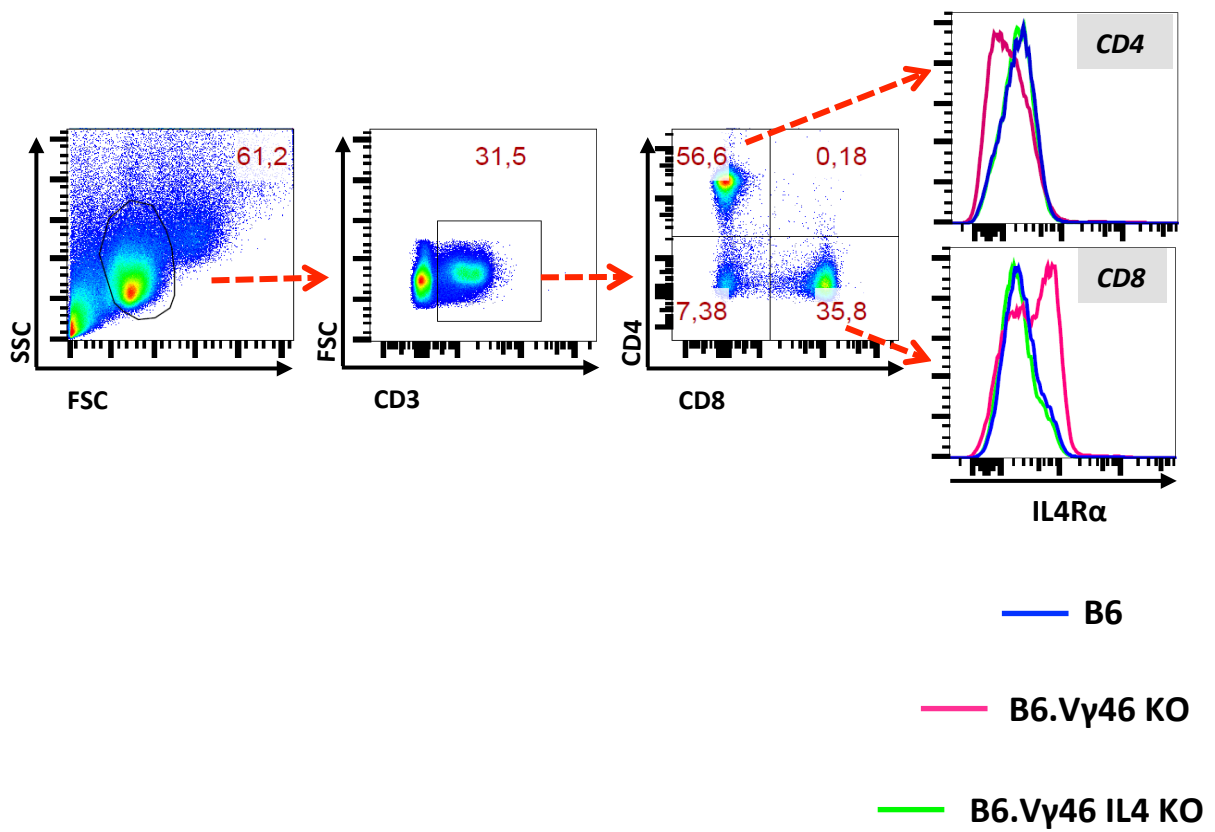

Supplement: S9 Fig — Splenocytes were stained and analyzed by flow cytometry as described in the Methods. (PDF) [file pone.0218827.s009.pdf]

S11 Fig.: Gating strategy for memory-phenotype CD4 and CD8 single-positive thymocytes

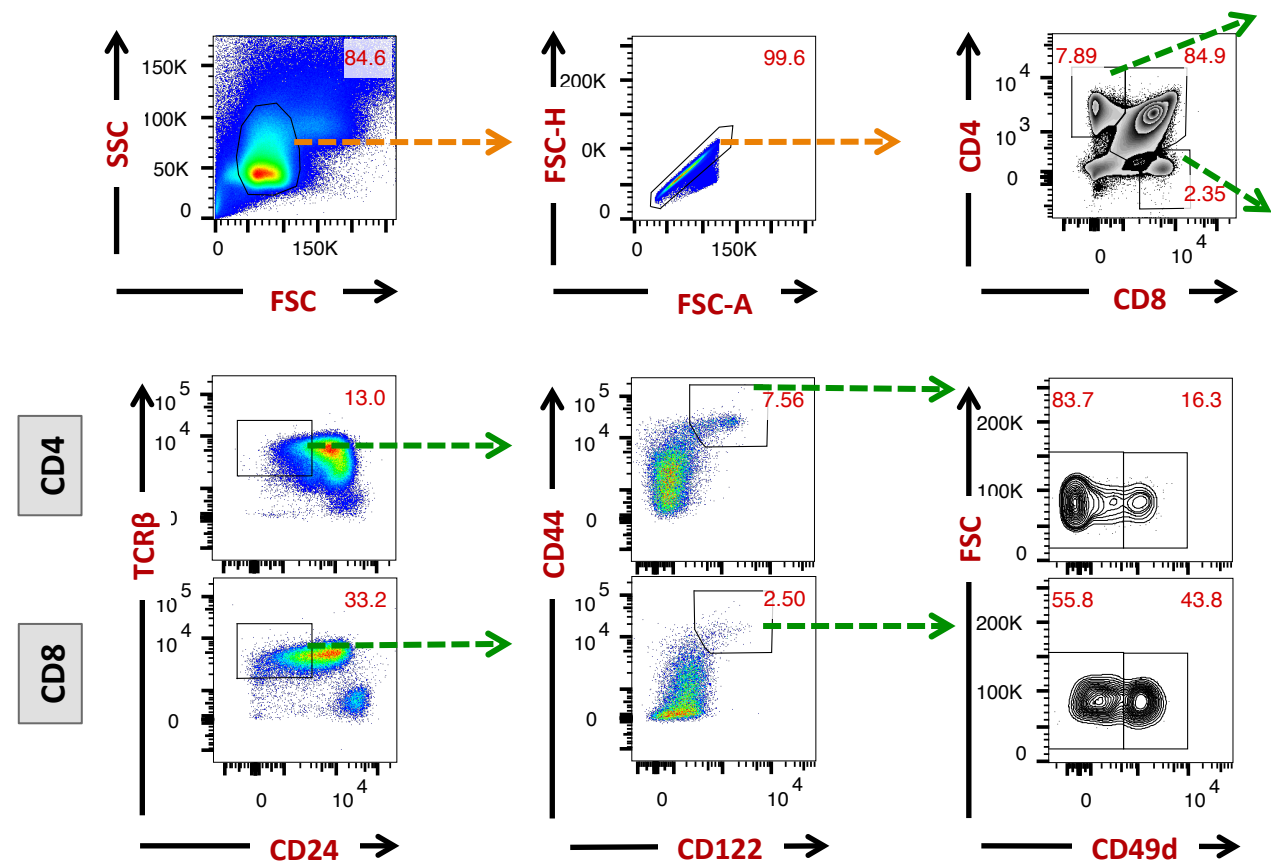

Supplement: S11 Fig — (PDF) [file pone.0218827.s011.pdf]
