# Supplementary material for: γδ T cells shape memory-phenotype αβ T cell populations in non-immunized mice
Source: PLoS One. 2019 Jun 25;14(6):e0218827. doi: 10.1371/journal.pone.0218827 (PMC6592556; doi:10.1371/journal.pone.0218827)

S3 Fig. : No significant effect of in vivo treatment with anti TCR mAbs on splenic B cells in C57BL/6 (wt) and B6.TCR-Vγ4/6 KO mice

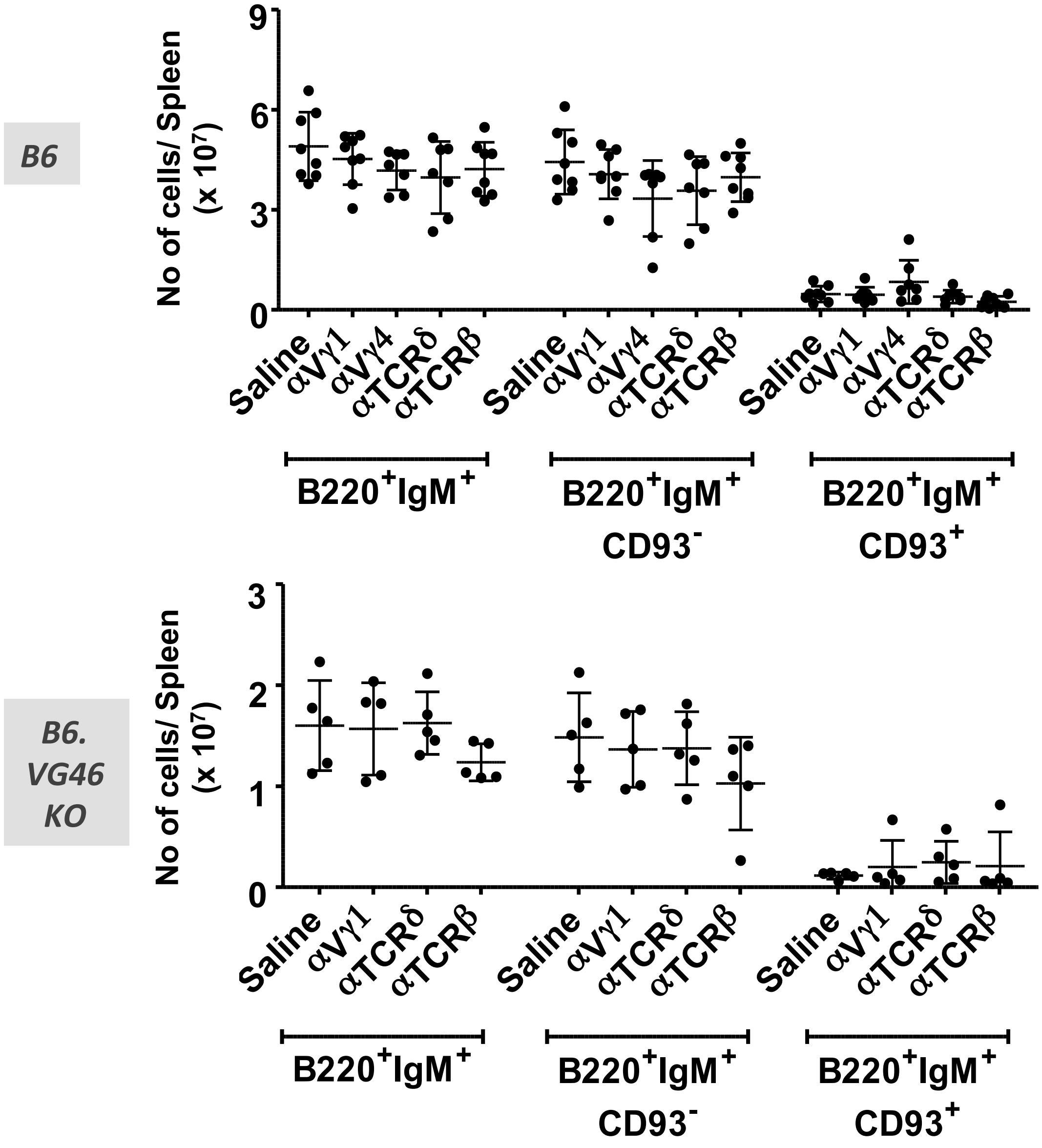

Supplement: S3 Fig — Adult mice were treated with i.v. injected anti TCR mAbs or saline alone, and analyzed by flow cytometry as detailed in the Methods. (PDF) [file pone.0218827.s003.pdf]
