# Supplementary material for: γδ T cells shape memory-phenotype αβ T cell populations in non-immunized mice
Source: PLoS One. 2019 Jun 25;14(6):e0218827. doi: 10.1371/journal.pone.0218827 (PMC6592556; doi:10.1371/journal.pone.0218827)
Supplement: S4 Fig — Female and male mice ages 8–12 wks were included in the comparisons shown (same mice as in Fig 1). ***p<0.001. (PDF) [file pone.0218827.s004.pdf]

S4 Fig
Relative frequencies of CD4+ and CD8+ αβ T cells in the spleen of γδ T cell-deficient mice

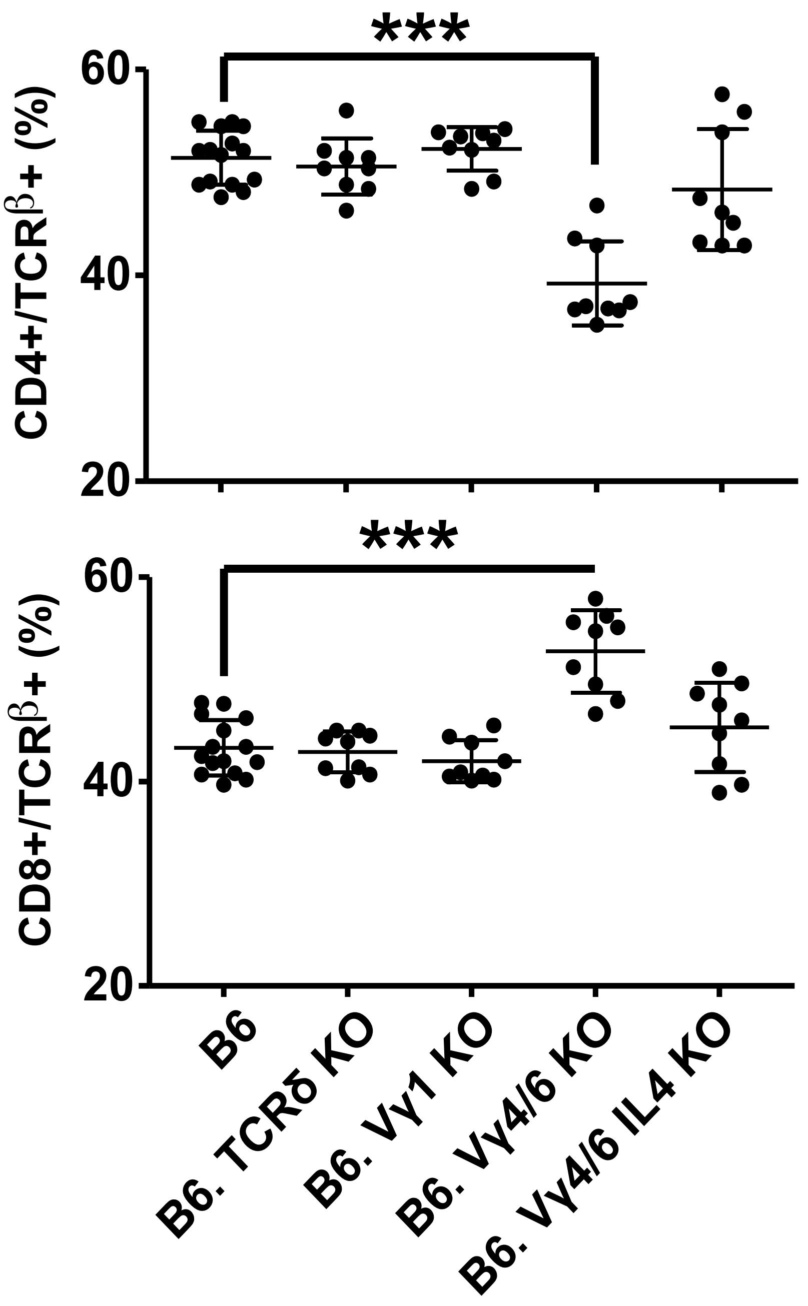

Female and male mice ages 8-12 wks were included in the comparisons shown (same mice as in in Fig 1). ***$p<0.001$
