# Supplementary material for: γδ T cells shape memory-phenotype αβ T cell populations in non-immunized mice
Source: PLoS One. 2019 Jun 25;14(6):e0218827. doi: 10.1371/journal.pone.0218827 (PMC6592556; doi:10.1371/journal.pone.0218827)

**S7 Fig.: Similar effect of γδ T cell-deficiencies on total splenic αβ T cells in female and male mice**

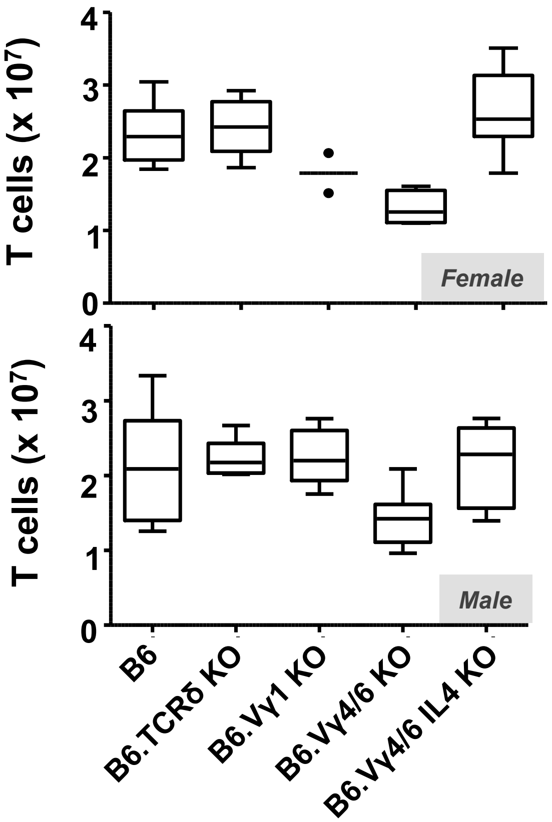

Supplement: S7 Fig — Comparison of total numbers of TCR-β+ cells in the spleen of C57BL/6 (B6), B6.TCRδKO, B6.TCR-Vγ1KO, B6.TCR-Vγ4/6KO, and B6.TCR-Vγ4/6KO/IL-4KO mice. Female and male mice ages 8–12 wks were included in the comparison shown in S6 Fig. n equal or greater than 10 mice/group. (PDF) [file pone.0218827.s007.pdf]
