# Supplementary material for: γδ T cells shape memory-phenotype αβ T cell populations in non-immunized mice
Source: PLoS One. 2019 Jun 25;14(6):e0218827. doi: 10.1371/journal.pone.0218827 (PMC6592556; doi:10.1371/journal.pone.0218827)

**S8 Fig.: Gating strategy for CD4+ and CD8+ memory-phenotype αβ T cells in the spleen of γδ T cell deficient mice**

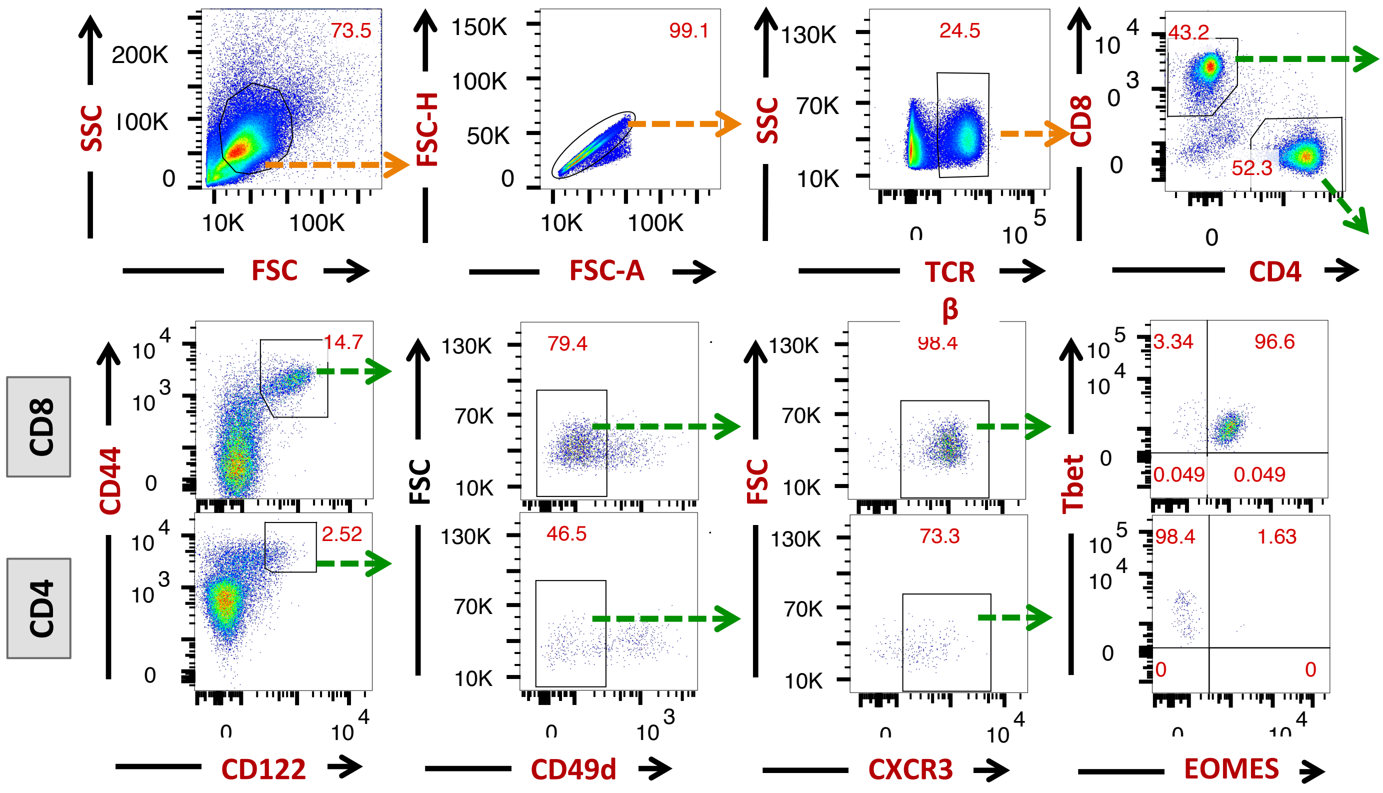

Supplement: S8 Fig — (PDF) [file pone.0218827.s008.pdf]
