# Supplementary material for: γδ T cells shape memory-phenotype αβ T cell populations in non-immunized mice
Source: PLoS One. 2019 Jun 25;14(6):e0218827. doi: 10.1371/journal.pone.0218827 (PMC6592556; doi:10.1371/journal.pone.0218827)

S10 Fig.: CD4+ and CD8+ memory-phenotype αβ T cells in the spleen of C57BL/6 (wt), B6.TCRδKO and B6.TCR-Vγ1KO mice

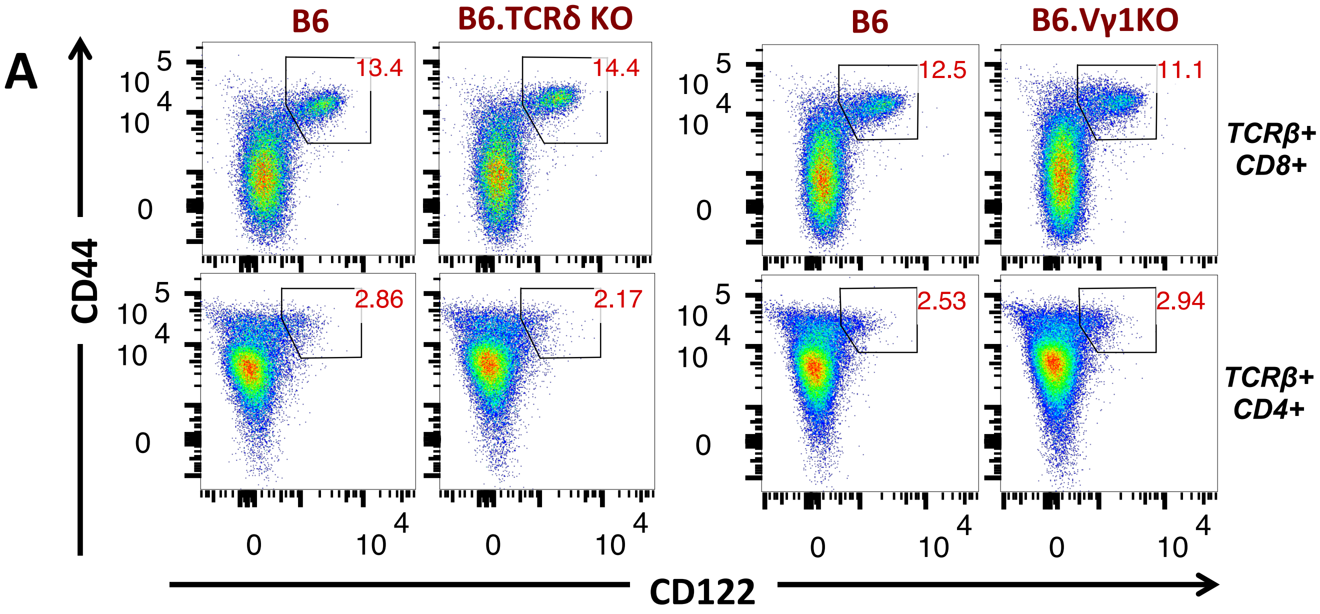

Supplement: S10 Fig — Unlike B6.TCR-Vγ4/6KO mice, no substantial changes in the frequencies of memory-phenotype αβ T cells were found in the spleens of B6.TCRδKO and B6.TCR-Vγ1KO mice. (PDF) [file pone.0218827.s010.pdf]
