# Supplementary material for: γδ T cells shape memory-phenotype αβ T cell populations in non-immunized mice
Source: PLoS One. 2019 Jun 25;14(6):e0218827. doi: 10.1371/journal.pone.0218827 (PMC6592556; doi:10.1371/journal.pone.0218827)
Supplement: S12 Fig — Male and female mice, ages 8–12 wks were included (same mice as in Fig 4). n equal or greater than 7 mice/group. *p<0.05, **p<0.01. (PDF) [file pone.0218827.s012.pdf]

S12 Fig
Relative frequencies of CD4+/CD8+, CD4+ and CD8+ thymocytes in γδ T cell-deficient mice

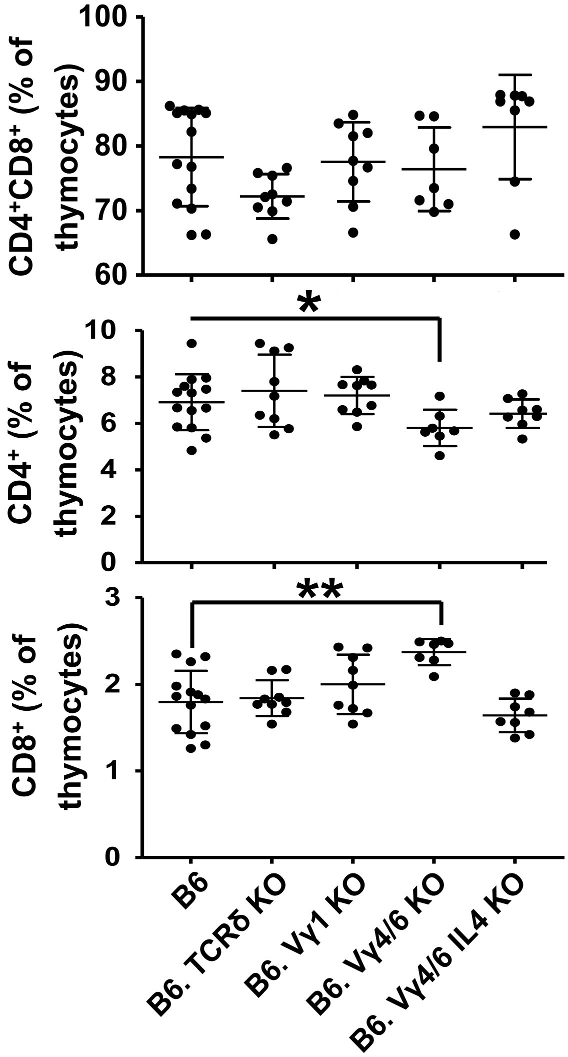

Male and female mice, ages 8-12 wks were included (same mice as in Fig.4). n equal or greater than 7 mice/group. *p<0.05, **p<0.01
